# Mad Is Required for Wingless Signaling in Wing Development and Segment Patterning in *Drosophila*

**DOI:** 10.1371/journal.pone.0006543

**Published:** 2009-08-06

**Authors:** Edward Eivers, Luis C. Fuentealba, Veronika Sander, James C. Clemens, Lori Hartnett, E. M. De Robertis

**Affiliations:** Howard Hughes Medical Institute, Department of Biological Chemistry, University of California Los Angeles, Los Angeles, California, United States of America; The University of Hong Kong, China

## Abstract

A key question in developmental biology is how growth factor signals are integrated to generate pattern. In this study we investigated the integration of the *Drosophila* BMP and Wingless/GSK3 signaling pathways via phosphorylations of the transcription factor Mad. Wingless was found to regulate the phosphorylation of Mad by GSK3 in vivo. In epistatic experiments, the effects of Wingless on wing disc molecular markers (*senseless*, *distalless* and *vestigial*) were suppressed by depletion of Mad with RNAi. Wingless overexpression phenotypes, such as formation of ectopic wing margins, were induced by Mad GSK3 phosphorylation-resistant mutant protein. Unexpectedly, we found that Mad phosphorylation by GSK3 and MAPK occurred in segmental patterns. Mad depletion or overexpression produced Wingless-like embryonic segmentation phenotypes. In *Xenopus* embryos, segmental border formation was disrupted by Smad8 depletion. The results show that Mad is required for Wingless signaling and for the integration of gradients of positional information.

## Introduction

Cells in the embryo are subjected to a multitude of growth factor signals that must be integrated to generate particular cell differentiation decisions. In the vertebrates, Smad1/5/8 provides a node of signaling integration. Smad1/5/8 are transcription factors activated by phosphorylation at the carboxy-terminus (Cter) by Bone Morphogenetic Protein Receptors (BMPR) [1]. In addition, Mitogen Activated Protein Kinase (MAPK) is able to phosphorylate the middle (linker) region of the protein, inhibiting BMP-Smad activity [2]. Work in amphibian embryos has shown that the neural inducing activity of Fibroblast Growth Factor 8 (FGF8) and Insulin-like Growth Factor (IGF) is mediated by inhibitory MAPK phosphorylations that decrease the activity of Smads [3]. Mouse fibroblasts carrying MAPK phosphorylation-resistant Smad1 (by homologous knock-in recombination) are resistant to the inhibitory effects of FGF in a BMP reporter assay [4]. Thus, BMP-Smads transduce MAPK signals.

Recently, it was discovered that the MAPK linker phosphorylations serve as primers for phosphorylations by Glycogen Synthase Kinase 3 (GSK3), which are essential for the polyubiquitinylation of Smad1 [5]. The Smad1 Cter phosphorylation by BMP receptor is followed by sequential MAPK and GSK3 phosphorylations, transport along microtubules to the centrosome, polyubiquitinylation, and degradation by proteasomes [5]–[6]. Inhibition of GSK3 or MAPK activity causes an increase in the duration of the BMP signal [5]. As will be seen below, MAPK and GSK3 also regulate activity independently of Cter phosphorylation in *Drosophila*.

**Figure 1:**
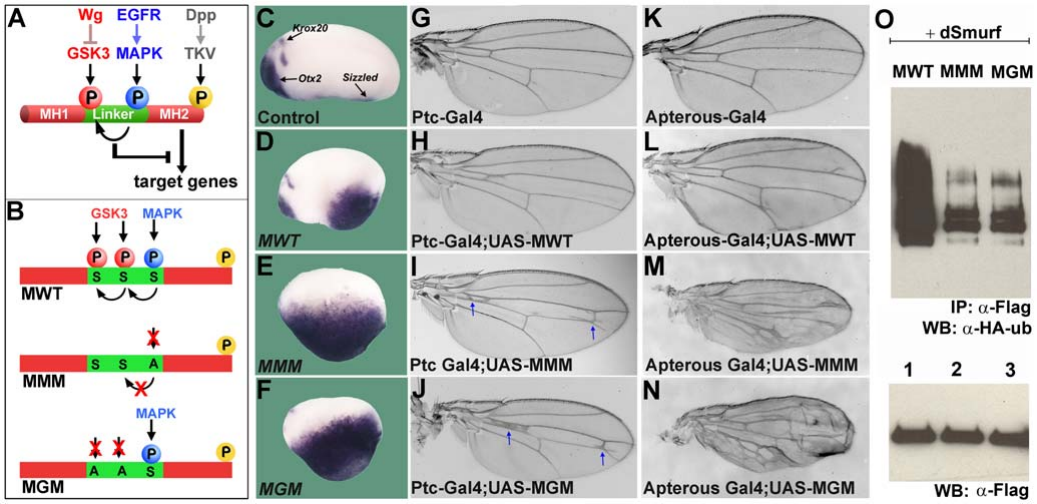
Phosphorylation-Resistant Mad Proteins are Hyperactive. (A) Model summarizing the integration of Dpp, EGFR and Wg signaling at the level of Mad phosphorylations in *Drosophila*. (B) Diagrams of Mad Wild Type (MWT), Mad MAPK Mutant (MMM) and Mad GSK3 Mutant (MGM) proteins. (C–F) Microinjection of *MMM* and *MGM* mRNAs into *Xenopus* embryos had stronger ventralizing activity than MWT, causing upregulation of *sizzled* (n = 17, 32, 26, and 30, two independent experiments). Brain markers o*tx2* and *krox20* were repressed. (G–J) Driving MMM and MGM with patched-Gal4 in the anterior wing compartment caused formation of ectopic crossvein-like tissue. This tissue links longitudinal veins two and three in both proximal and distal regions, pulling the two veins closer together. (K–N) Driving phosphorylation-resistant Mads with apterous-Gal4 induced ectopic vein tissue and blistering, indicating increased Dpp signaling. (O) Polyubiquitinylation of Mad requires GSK3 and MAPK phosphorylation sites. Lane 1, 293T cells cotransfected with MWT-Flag, *Drosophila* Smurf and HA-ubiquitin all cloned in pCS2. The strong smear represents polyubiquitinylated Mad tagged with HA-ubiquitin. Lanes 2 and 3, polyubiquitinylation was greatly decreased in the MMM and MGM mutant proteins. The lower panel shows equal levels of immunoprecipitated Mad (α-Flag).

Proteasomal degradation of Smad1 is a major regulator of BMP signal termination [4]–[6]. GSK3 function, at least for β-catenin phosphorylations, can be regulated by Wnt signaling [7]–[8], and therefore the GSK3 sites in Smads offer the possibility of integrating three of the main signaling pathways – BMP, MAPK and Wnt - on a single molecule (Figure 1A). In *Xenopus*, we showed that Wnt induced epidermis in dissociated ectodermal cells, and that this activity was blocked by overexpressing a dominant-negative Smad5 construct [5]. This suggested a new branch of the canonical Wnt pathway signaling through Smad1 phosphorylation at GSK3 sites which, surprisingly, was found to have a complete requirement for β-Catenin [5].

Integrating Wnt and BMP signaling is crucial in developmental biology, for it has been shown that a gradient of Wnt is a major determinant of the antero-posterior (A–P) axis, with low levels causing head and high levels tail development [9]. Dorsal-ventral (D–V) cell differentiation decisions are regulated by a gradient of BMP [10]–[11], and integration of Wnt at the level of BMP-Smads could explain how A-P and D-V pattern are seamlessly integrated when development is challenged experimentally [12]. The *Drosophila* genome contains a single BMP-Smad, called *mothers against dpp* (Mad) [13], which has a single canonical MAPK/Erk phosphorylation site (PXSP) and two GSK3 (SXXXSp) sites upstream of it. The fruit fly therefore offered an excellent system to investigate signaling integration.

The present study was initiated to test whether endogenous Mad was required for Wingless (Wg) signaling in *Drosophila*. Novel reagents were generated, such as phospho-specific antibodies for pMad^GSK3^ and pMad^MAPK^, and Mad RNAi knockdown constructs that can specifically inhibit maternal or zygotic *Mad* mRNA. Mutant forms of Mad resistant to GSK3 phosphorylation, which mimic Mad receiving a maximal amount of Wg, were hyperactive and caused typical Wg-like overexpression phenotypes [14] in wing clonal analyses, such as ectopic sensory bristles and wing margin duplications. Mad RNAi clones eliminated the wing margin. In the larval wing disc, Mad knockdown with RNAi inhibited the increases in *senseless*, *optomotor blind*, *distalless* and *vestigial* transcripts caused by Wg. Overexpression of GSK3-resistant Mad or Wg protein generated similar phenotypes. Thus, Mad was found to be required for Wg signaling in vivo.

Unexpectedly, we discovered a novel role for Mad during segment formation. The endogenous pMad^MAPK^ antigen was stabilized, and nuclear pMad^GSK3^ inhibited, in regions overlapping with Wg segmental expression in wild type embryos. Mad knockdown caused Wg-like loss-of-function phenotypes in embryonic cuticles, and overexpression of GSK3-resistant Mad caused naked cuticle, mimicking Wg gain-of-function phenotypes. These findings may have important implications for the integration of patterning signals. In addition, we report that in *Xenopus laevis* Smad8 morpholinos prevent somite border formation, which may have evolutionary implications.

## Results

### Mad Mutants Resistant to MAPK and GSK3 Phosphorylation Are Hyperactive

We first asked whether the MAPK and GSK3 phosphorylation sites in the linker region of *Drosophila* Mad were important in modulating its C-terminal BMP activity (Figure 1A). Serines in the single MAPK site or in the two GSK3 sites upstream of it were mutated into alanines, and designated Mad MAPK Mutant (MMM) and Mad GSK3 Mutant (MGM) (Figure 1B). To test these phosphorylation-resistant Mad constructs, mRNAs were microinjected into *Xenopus* embryos. Both *MMM* and *MGM* expanded the BMP-dependent marker *sizzled* into more dorso-lateral regions and reduced forebrain (*otx2*) and midbrain (*krox20*) markers when compared to microinjection of *MWT* (Figure 1C–1F).

Mad transgenic flies in the UAS vector [15] were generated and driven in the anterior wing compartment using a patched-Gal4 driver. Expression of MMM and MGM, but not MWT, induced a crossvein-like phenotype (Figure 1G–1J, arrows; Figure S1). When driven in the dorsal wing compartment with apterous-Gal4, MMM and MGM induced large amounts of ectopic vein tissue, accompanied by blistering (Figure 1K–1N). The excessive wing vein tissue can be a sign of increased BMP signaling. The phosphorylation of Mad by MAPK and GSK3 is required for its efficient polyubiquitination and degradation (Figure 1O; [6]) We conclude from these experiments that inactivation of the MAPK or GSK3 phosphorylation sites resulted in hyperactive Mads, causing increased duration of Dpp/BMP signals, most likely through a decrease in the rate of Mad degradation.

### Phospho-resistant Mad Mutants display Wg-like phenotypes

**Figure 2:**
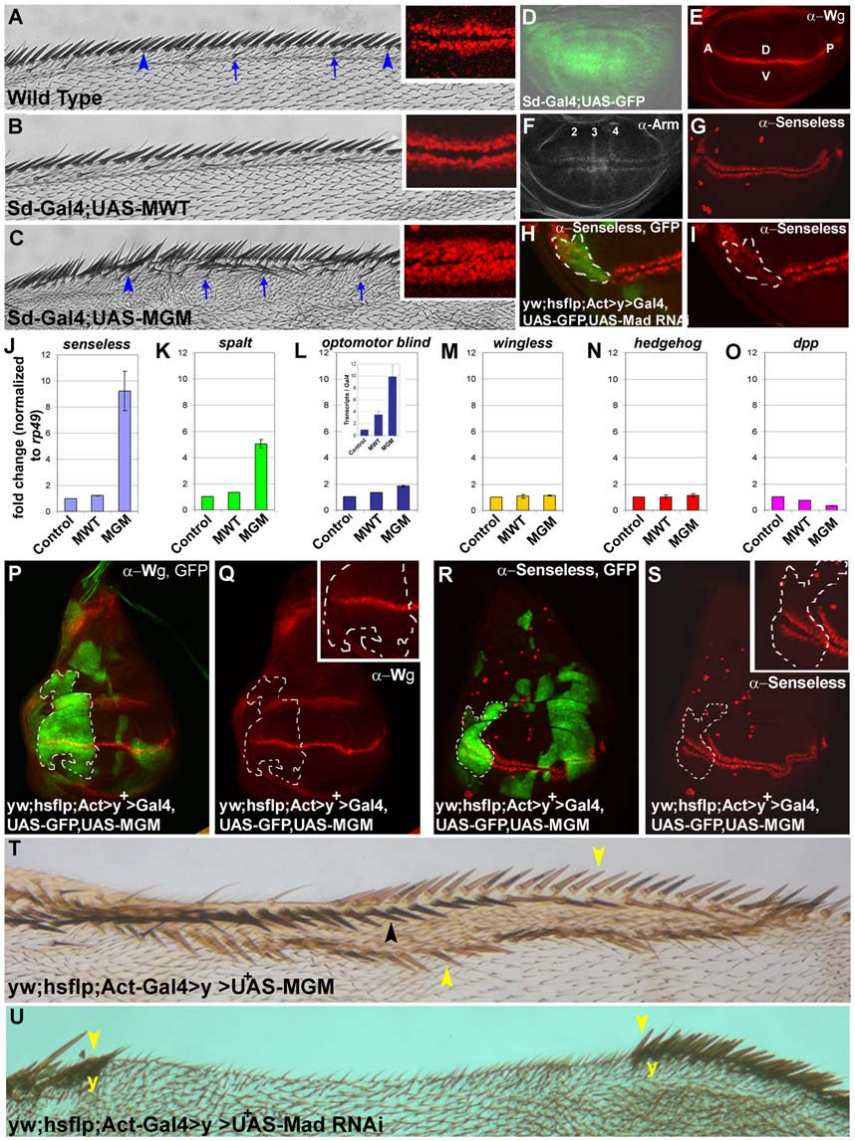
Mad GSK3 Mutants Mimic Wg Overexpression. (A–C) Additional bristles are induced in the wing margin by MGM driven by scalloped-Gal4. Arrows indicate chemosensory bristles and arrowheads stout mechanosensory bristles. Insets show that the number of Senseless-expressing bristle precursors in wing imaginal discs is increased by MGM, but not MWT.(D–G) Expression domains of sd-Gal4 driver, Wg protein, Armadillo stabilized by Wg (expression in the proveins is noted), and Senseless in wing discs. (H and I) Senseless protein expression was inhibited in Mad RNAi clones marked by GFP. (J–O) Quantitative RT-PCR of wing discs showing that MGM increased both a Wg target gene (*senseless*) and Dpp target genes (*spalt* and *optomotor blind*), while not affecting *wg* or *hh* levels. *dpp* was inhibited by MGM RNA. Samples were normalized for *rp49*, except for the inset in L in which *Gal4* mRNA was used. (P and Q) Clonal overexpression of MGM does not change Wg levels. (R and S) MGM clone in the anterior margin causes ectopic expression of Senseless within the clone (inset). (T) Overexpression of MGM in clones marked by *yellow* induced duplications of the wing margin (n = 24). Yellow arrowheads indicate ectopic margins and a black arrowhead the wild type one. (U) Knockdown of Mad in clones partially eliminates the wing margin In this large clone the remaining margin bristles are *yellow* (*y*).

We next investigated whether stabilized MGM phenocopied Wg signaling, which normally induces sensory bristles along the wing margin [14]. When MGM was driven with either scalloped-Gal4 (sd-Gal4) or A9-Gal4, additional sensory bristles, both stout mechanosensory (arrowheads) and chemosensory bristles (arrows), were formed in the wing margin (compare Figure 2A and 2C), while overexpression of MWT had little effect (Figure 2B). Overexpression of MMM using A9-Gal4 driver induced ectopic bristles on longitudinal veins (Figure S2). When driven by Sd-Gal4, both MGM and MMM could induce chemosensory bristles on the wing blade itself (Figure S2). This ectopic bristle formation in the wing blade induced by mutant Mad proteins occurred in the absence of ectopic vein tissue, suggesting that the Mad Wg-like phenotypes can occur at low levels of pMad^Cter^ signals. To confirm this hypothesis, we overexpressed Dpp in the wing margin, which was able to increase wing size, but did not induce ectopic bristles (Figure S3). Taken together, the results on ectopic induction of bristles suggest that both MGM and MMM generate Wg-like phenotypes when overexpressed.

Sd-Gal4 is driven as a gradient in the wing pouch by a stripe of Wg which stabilizes Armadillo/β-Catenin in its flanking regions (Figure 2D–2F). Bristle formation requires the transcription factor Senseless, which is expressed in the vicinity of the Wg stripe that marks the wing margin (Figure 2G). MGM increased the number of cells expressing Senseless (Figure 2A–2C insets; Figure S4), as well as the size of the wing pouch. Dpp overexpression has been shown to increase the size of the wing pouch [16]. MGM induced Senseless in regions close to the stripe of Wg, but not over the entire wing pouch, suggesting that it requires additional co-factors such as stabilized Armadillo/β-Catenin (Figure 2F). This would agree with results in *Xenopus* showing that β-Catenin is required for the regulation of Smad1 by the Wnt pathway [5].

The levels of *senseless* mRNA, a target of Wg, in wing discs were increased by MGM, but not by MWT (Figure 2J). MGM also increased the Dpp target genes *spalt* and *optomotor blind* [16], without increasing the levels of *wg*, *hedgehog* or *dpp* (Figure 2K–2O). Similar conclusions were reached whether the quantitative RT-PCR were normalized with ribosomal protein 49 (*rp49*) or *Gal4* transcripts (Figure 2L, inset).

We next analyzed MGM flp-out clones marked by GFP in the wing disc or by *yellow* (*y*) bristles in the adult [17]. It was found that clones overexpressing MGM did not increase Wg expression (Figure 2P and 2Q), yet were able to cause duplications of the wing margin, a typical Wg overexpression phenotype (Figure 2T). Clones in the anterior disc margin caused formation of ectopic rows of Senseless-expressing bristle precursor cells within the clone (Figure 2R and 2S, see inset). Conversely, knockdown of Mad with RNAi (see below) caused decreased Senseless expression within clones (Figure 2H and 2I), which were accompanied in the adult wing by losses of the anterior wing margin within clones (Figure 2U). Mad RNAi clones phenocopy Wg loss-of-function phenotypes [14]. MWT was without effect in these clonal studies, and MGM clones did not affect Engrailed or *hh-LacZ* expression (Figure S5 and S10).

We conclude from these studies on the wing margin that overexpression of these mutant proteins mimic Mad receiving a maximal possible dose of Wg, causing Wg-like phenotypes in the absence of increased Wg signals. Conversely, Mad depletion caused Wg loss-of-function phenotypes. These data support the molecular pathway proposed in Figure 1A, in which Mad phosphorylation is regulated by Wg signal transduction.

### pMad^GSK3^ Is Decreased and pMad^MAPK^ Stabilized by Wg

**Figure 3:**
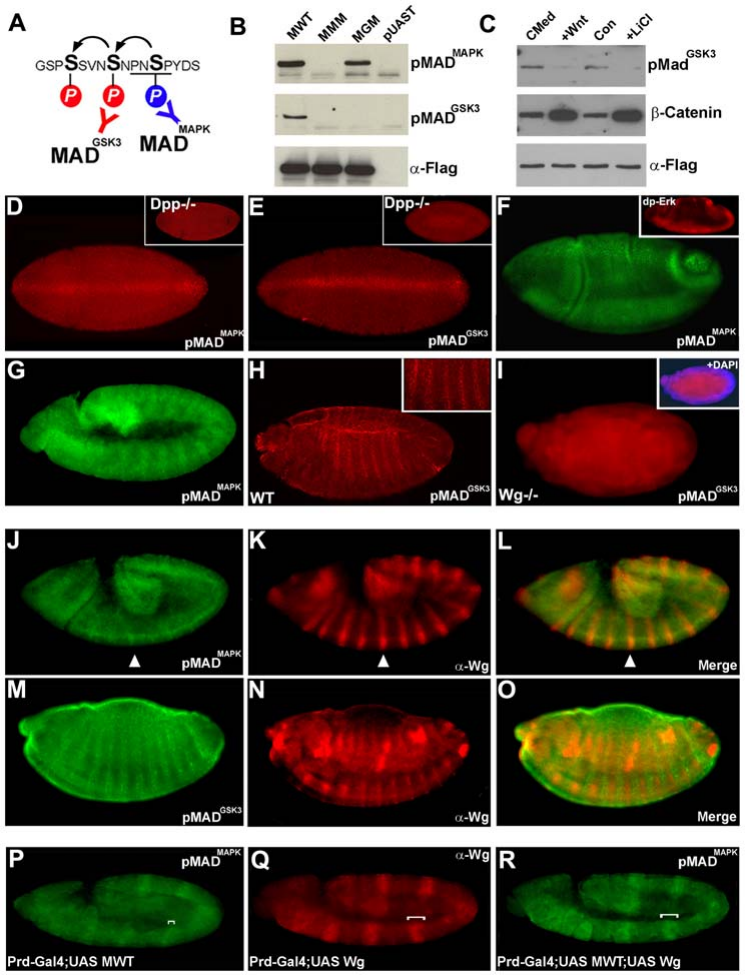
Phospho-Specific Antibodies Reveal Wg-regulated Segmental Expression Patterns of pMad^MAPK^ and pMad^GSK3^. (A and B) Western blot analysis of pMad^MAPK^ and pMad^GSK3^ antibodies demonstrating that they were phospho-specific, and that GSK3 phosphorylation had an absolute requirement for MAPK priming. *Drosophila* S2 cells were transiently transfected with the plasmids indicated. (C) Cultured 293T cells stably transfected with Mad-Flag treated with L-cell control conditioned medium (CMed), Wnt3a medium, control DMEM (Con), or 30 mM LiCl in DMEM for 2 hours. Wnt3a and LiCl inhibited the Mad^GSK3^ phosphorylation band and increased β-Catenin levels (indicating that the Wnt treatment was effective). (D and E) pMad^MAPK^ and pMad^GSK3^ antibodies stain the entire blastoderm and a Dpp-dependent dorsal stripe (inset). (F) pMad^MAPK^ tracks ventral EGFR-activated MAPK (inset shows diphospho-Erk staining). (G and H) Segmental staining of pMad^MAPK^ (Stage 9) and pMad^GSK3^ (Stage 17). (I) In Wg null mutants segmental expression is lost. Mutant embryos were identified by lack of staining with Wg antibody. Inset shows same embryo stained with DAPI to indicate that, despite its abnormal shape, it reached late stages of development. (J–L) Wg stabilizes pMad^MAPK^, overlapping with Wg stripes. (M–O) Nuclear pMad^GSK3^ accumulates in between Wg stripes, indicating that Wg inhibits Mad phosphorylation at GSK3 sites in vivo. (P–R) Wg overexpression driven with prd-Gal4 stabilizes pMad^MAPK^ over a broader domain compared to just MWT alone (compare brackets in P and R). This experiment shows that Wg expression stabilizes pMAD^MAPK^.

To determine whether the MAPK and GSK3 sites in Mad were phosphorylated in vivo, we generated phospho-specific antibodies (Figure 3A). The anti-pMad^GSK3^ antibody did not recognize MGM (as expected for a phospho-specific antibody) or MMM, indicating an obligatory requirement for the priming MAPK phosphorylation (Figure 3B). In cells stably transfected with Mad-flag, the addition of L-cell Wnt3a conditioned medium, or of the GSK3 inhibitor Lithium chloride, caused a decrease in the Mad band phosphorylated by GSK3, indicating that Wnt signaling can inhibit this phosphorylation (Figure 3C). These rabbit antibodies failed to stain *Drosophila* wing imaginal discs specifically.

In *Drosophila* embryos, both pMad^MAPK^ and pMad^GSK3^ stained the entire cellular blastoderm, with stronger nuclear accumulation along a dorsal stripe, which did not form in *Dpp* null embryos (Figure 3D and 3E). Staining in the rest of the blastoderm was Dpp-independent, and pMad^MAPK^ stained a single cytoplasmic spot of antigen usually adjoining one of the centrosomes (Figure S6), which marks Mad targeted for degradation [6]. The persistence of the asymmetric centrosome-associated spots in Dpp mutants indicates that MAPK and GSK3 phosphorylations can occur independently of Dpp. At early gastrula, pMad^MAPK^ and pMad^GSK3^ tracked diphospho-Erk/EGFR activity [18], particularly in the ventral region of the embryo (Figure 3F, see inset) where Dpp signaling is low. Thus, linker phosphorylations can occur independently of Dpp signaling.

Importantly, at late segmentation stages pMad^MAPK^ and pMad^GSK3^ antigens displayed segmental expression patterns (Figure 3G and 3H). The pMad^MAPK^ striped pattern was seen during band elongation, whereas pMad^GSK3^ stripes were more distinct later, at germ band retraction. Detailed analyses revealed that these bands were non-overlapping. Double stainings showed that pMad^MAPK^ stripes overlapped with Wg protein, while nuclear pMad^GSK3^ staining was stronger in between Wg stripes (Figure 3J–3O). These observations in wild type embryos show that Wg inhibits Mad GSK3 phosphorylation, causing accumulation of pMad^MAPK^ antigen in regions where Wg is high because it decreases degradation of Mad (see Figure 1A). Importantly, these studies on wild type embryos show that Mad phosphorylation by GSK3 is indeed regulated in vivo.

Mutation of Wg caused the pMad^GSK3^ stripes to disappear (Figure 3I). In gain-of-function experiments, Wg driven by a *paired*-Gal4 driver expanded the area stained by pMad^MAPK^ antibody in every other segment (Figure 3P–3R, see brackets). This is consistent with the view that Wg prolongs the duration of Mad/Smad1 signal by decreasing the rate of degradation of Mad and pMad^MAPK^ [5]. We conclude from these antibody studies that endogenous Mad is phosphorylated at MAPK and GSK3 sites in *Drosophila* embryos, and that this process is regulated by Wnt signals. The most interesting finding was that segment formation might be regulated by Mad linker phosphorylations.

### Depletion of Mad by RNAi

**Figure 4:**
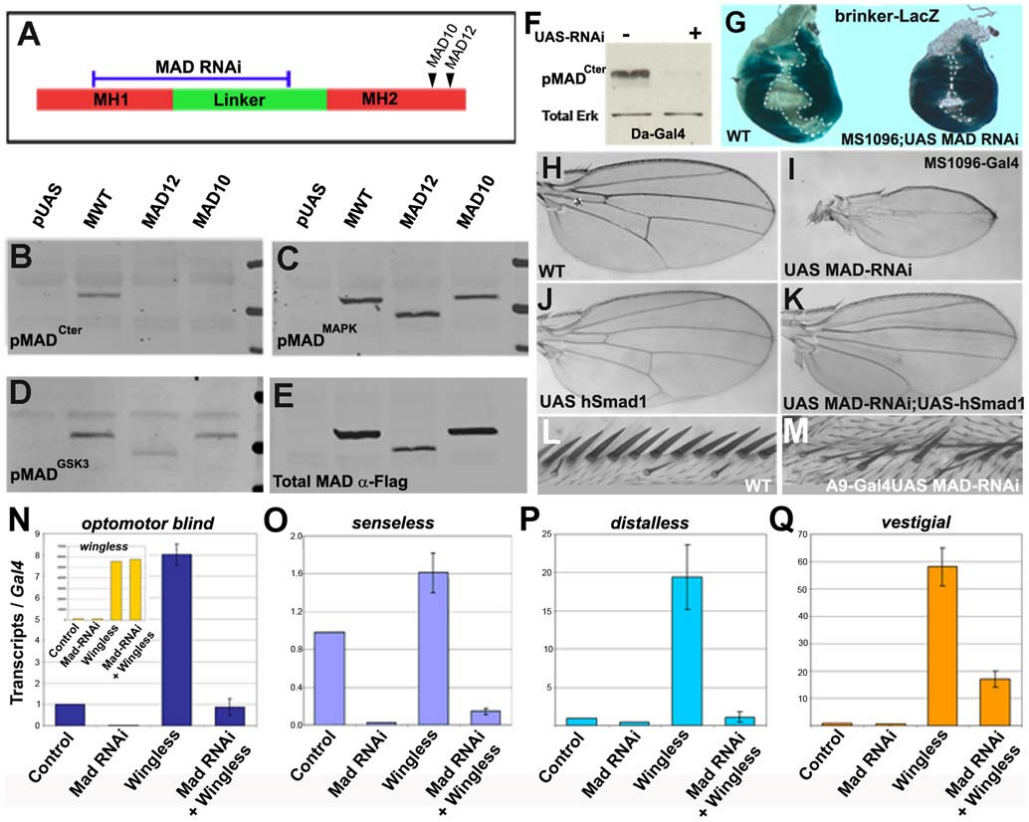
Mad^10^ and Mad^12^ Alleles Are not Nulls; Mad RNAi Is an Effective and Specific Loss-of-Function Reagent. A) Schematic representation of Mad, showing RNAi and mutant sites. (B–E) Mad^10^ and Mad^12^ mutants are phosphorylated in the linker region in the absence of C-terminal phosphorylation. (F) UAS-Mad RNAi depleted stage 15 embryos of endogenous pMad^Cter^ when driven by daughterless-Gal4. (G) Repression of brinker-LacZ reporter by Dpp (demarcated by hatched lines) was inhibited by Mad RNAi in wing imaginal discs. (H–K) Mad RNAi driven by MS1096-Gal4 causes complete vein loss at room temperature, which was rescued by UAS-hSmad1. (L and M) Anterior margin mechanosensory bristles are lost when two copies of Mad RNAi were driven with A9-Gal4; this phenocopies Wg loss-of-function. (N–Q) Epistasis by QRT-PCR showing that Wg overexpression in the wing pouch, driven by sd-Gal4, increased transcript levels of the reporter genes *optomotor blind*, *senseless*, *distalless* and *vestigial*, and that this induction required Mad Samples were normalized for Gal4 mRNA levels Inset shows that levels of Wg transcripts were not affected by Mad RNAi.

The phosphorylation patterns of pMad^MAPK^ and pMad^GSK3^ suggested that linker phosphorylation could function during segmentation (Figure 3L and 3O). However, larvae carrying the “null” mutations Mad^10^ and Mad^12^ are perfectly segmented and die at third instar [13]. These alleles are caused by missense and nonsense mutations, respectively, and are located close to the C-terminus of Mad (Figure 4A). We reasoned that these mutations might impair Dpp C-terminal signaling but leave regulation by the EGFR/MAPK or Wg/GSK3 pathways intact.

When cDNAs encoding MWT, Mad^10^ or Mad^12^ were expressed in *Drosophila* S2 cells, we observed that MWT was detectable by phospho-Mad^Cter^ antibody, while both mutants were not (Figure 4B). However, the pMad^MAPK^ and pMad^GSK3^ sites were phosphorylated in the mutant proteins (Figure 4C and 4D). We conclude that the Mad^10^ and Mad^12^ proteins were stably translated (Figure 4E) and were nulls for Dpp C-terminal phosphorylation, but were still regulated by MAPK and GSK3 linker phosphorylations. In microinjected *Xenopus* embryos, *Mad^12^* mRNA reduced forebrain structures marked by *Rx2a* (Figure S7). When the GSK3 sites of Mad^12^ were mutated (mimicking a protein receiving a maximal Wnt signal) the head was almost eliminated (Figure S7C). Microinjection of Wnt10b DNA, a canonical Wnt, generated similar posteriorized phenotypes, but in addition increased levels of the ventral marker *sizzled*, presumably because it affects the stability of endogenous Smad1/5/8 after signaling by BMP4/7 (Figure S7C and D). These results suggest that Mad linker phosphorylations can occur independently of C-terminal phosphorylations mediated by BMP receptors, and that Mad can still function in signaling in the absence of C-terminal phosphorylation.

In order to deplete Mad transcripts in vivo, a fragment of the Mad sequence, including most of the MH1 domain and the linker phosphorylation sites (Figure 4A), was cloned into the pWiz RNAi vector, which can be driven by the Gal4/UAS system [19]. (An RNAi construct directed against the C-terminal domain of Mad gave similar phenotypes but was weaker, data not shown). Eight independent transgenic lines were tested and all showed Dpp-like loss-of-function wing phenotypes. Strains homozygous for transgenes in chromosomes 2 or 3 facilitated subsequent analyses, as 100% of the embryos expressed the Mad RNAi. Expression of Mad RNAi in embryos or S2 cells strongly inhibited Mad levels (Figures 4F and S8). In wing imaginal discs, the repression of the reporter brinker-LacZ by Dpp [20] was inhibited by Mad RNAi (Figure 4G).

Driving RNAi in the wing reduced its size and eliminated veins at room temperature (Figure 4H and 4I). Doubling the dose of RNAi, or driving Gal4 at higher temperatures, resulted in flies lacking wings (Figure S9). The RNAi effects were specific, because wing size and vein development were rescued by co-expression of a human Smad1 transgene (Figure 4H–4K). Human Smad1 was also able to rescue lethality of Mad RNAi driven by daughterless-Gal4. Mad RNAi pupae failed to eclose into adult flies (n = 1029), while in the presence of UAS-hSmad1 97% (n = 1055) were rescued and produced viable and fertile flies. We conclude that Mad RNAi is specific, with no off-target effects. Mad depletion also caused Wg loss-of-function phenotypes; when driven in the wing pouch with A9-Gal4, mechanosensory bristles were partially lost in the wing margin (Figure 4l and 4M). Mad RNAi provides a powerful new reagent that inhibits all aspects of Mad function, including its regulation by Wg/GSK3.

In quantitative RT-PCR analyses of wing discs driven by sd-Gal4, Mad RNAi inhibited expression of the classical Dpp target gene *optomotor blind* (*omb*, [16]) (Figure 4N). In addition, overexpression of Wg significantly increased *omb* transcript levels, and knockdown of Mad reduced this increase to wild type levels (Figure 4N). As a control, the levels of Wg mRNA overexpressed were determined and found to remain unchanged by the introduction of the UAS-Mad RNAi (Figure 4N, inset). Additional controls showed that these transcriptional effects on marker genes were not due to changes in *hedgehog* or *dpp* expression (Figure S10). All transcripts were normalized for Gal4 mRNA expression levels. Wg overexpression with sd-Gal4 increased expression of the Wg target gene *senseless* (Figure 4O). *Distalless* and *vestigial*, which respond mainly to Wg, but also to Dpp [16], [21], were markedly increased by Wg overexpression (Figure 4P and 4Q).

The effects of Wg on *optomotor blind*, *senseless*, *distalless* and *vestigial* transcripts were all inhibited by Mad RNAi (Figure 4N–4Q). Conversely, in a gain-of-function situation MGM overexpression was able to activate the Wg target *senseless* (Figure 2J). Taken together, these epistatic loss- and gain-of-function experiments support the view that the Wg signal requires Mad.

### Mad is required for Wg signaling during neurogenic ectoderm differentiation

**Figure 5:**
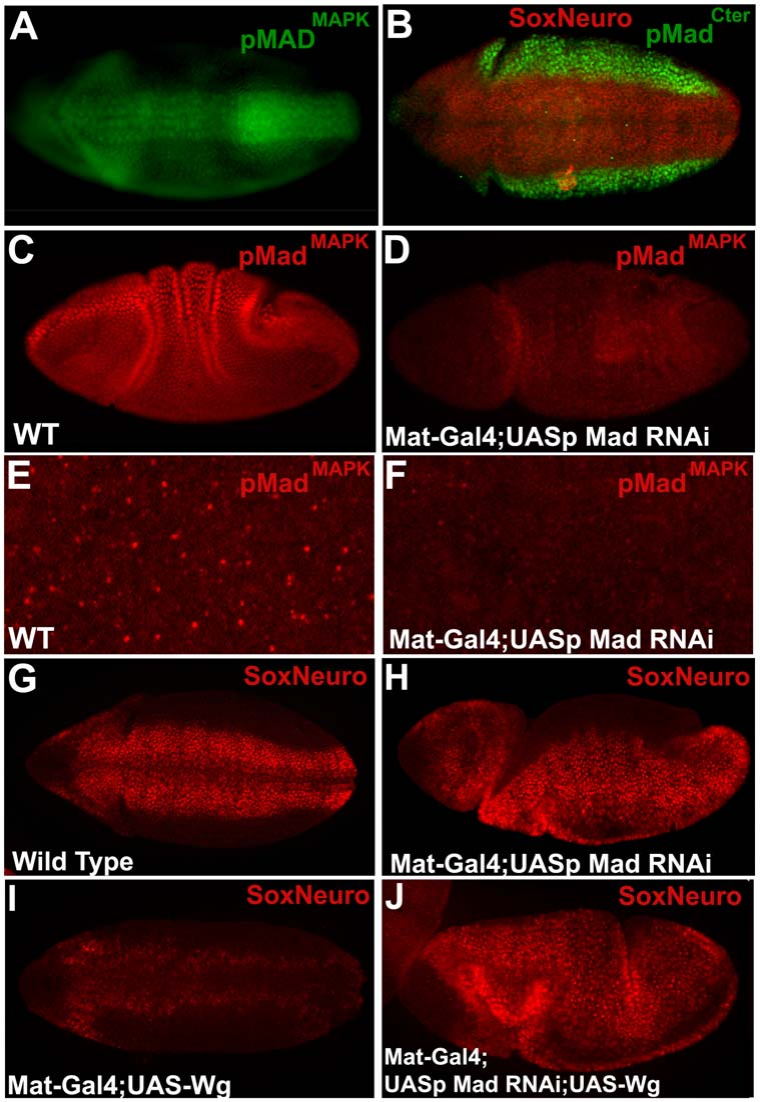
Mad Is Epistatic to Wg Signaling During Neurogenic Induction. (A) Mad was phosphorylated at its MAPK sites in developing CNS neuroblasts. (B) pMad^Cter^ was excluded from the neurogenic ectoderm marked by SoxNeuro (stage 8). (C and D) Mad RNAi driven in the egg by pUASp knocked down pMad^MAPK^ staining (stage 7). (E and F) Maternal Mad RNAi knocked down pMAD^MAPK^ centrosomal staining (stage 7, see Figure S5 for asymmetric centrosomal staining). (G–J) Mad RNAi increased neurogenic ectodermal nuclei marked by SoxNeuro at stage 8, Wg overexpression reduced it, and the double Wg;RNAi embryos displayed the Mad depletion phenotype. All images were taken at identical exposure conditions. This experiment shows that Mad is epistatic to Wg.

A role of Mad in neurogenic ectoderm differentiation was suggested by the finding that pMad^MAPK^ antibody stained brightly the developing central nervous system (CNS) (Figure 5A). At germ band extension, pMad^Cter^ was excluded from this neurogenic region, which is marked by SoxNeuro (Figure 5B; [22]). These results show that Mad linker phosphorylations can also occur independently of BMP-induced Mad C-terminal signaling in *Drosophila*. In Dpp nulls, the neurogenic ectoderm marker SoxNeuro, was expressed ectopically throughout the embryo ([22] and our data not shown), indicating that Mad signaling may regulate the decision between differentiating neurogenic or non-neurogenic ectoderm in *Drosophila*, as Smad1/5/8 does in vertebrates [6], [23].

To induce neurogenic tissue by Mad RNAi it was necessary to inhibit the maternal *Mad* mRNA stockpile. Because the pUAST promoter is not transcribed in oocytes, we recloned Mad RNAi into the pUASp vector, which is transcribed maternally from a p-element promoter [24]. When driven in the egg, maternal Mad RNAi caused a marked decrease of Mad protein by gastrula stage (Figure 5C–5F). SoxNeuro marks neurogenic ectoderm nuclei at germ band elongation, and Mad RNAi expanded this tissue (Figure 5G and 5H). Overexpression of Wg (introduced by the sperm into eggs containing maternal Gal4) at early embryonic stages, decreased the number of neurogenic ectodermal cells marked by SoxNeuro (compare Figure 5I to 5G). When Wg and Mad RNAi were co-expressed ectopic neurogenic ectoderm was still present (Figure 5J). Thus the Mad depletion phenotype was epistatic to Wg overexpression, showing that Mad is required for the reduction in neurogenic ectoderm (Figure 5G–5J) caused by Wg. These epistatic experiments support the view that Mad is required for Wg to signal during early neurogenesis in *Drosophila* embryos.

### *Drosophila* Mad Is Required for Segmental Patterning

**Figure 6:**
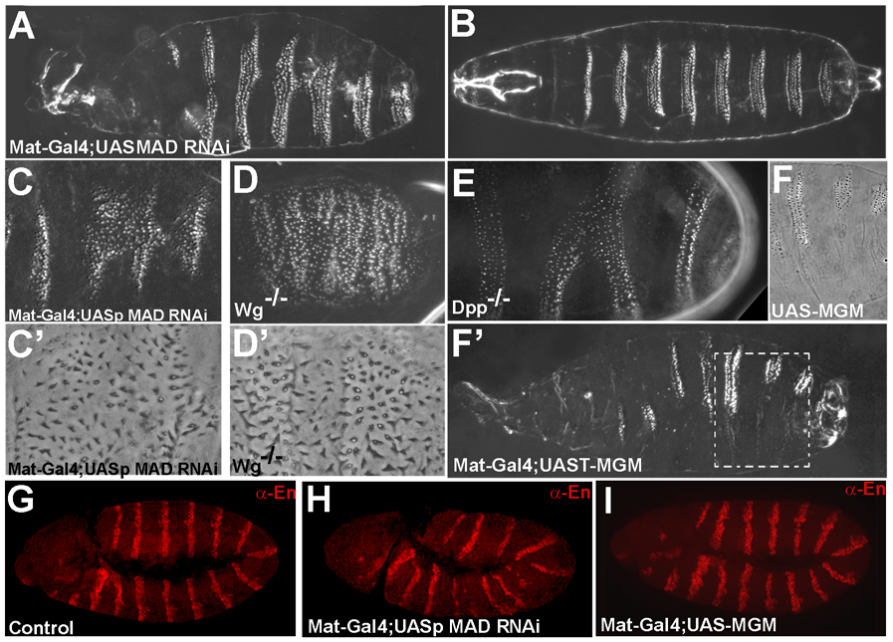
Mad Is Required for Segmentation in *Drosophila*. (A–B) Early depletion of Mad caused wider (ventralized) denticle belts and internalized posterior spiracles in embryonic cuticles (n = 259 cuticles, 20% ventralized and 34% ventralized with denticle belt fusions). (C and C') Denticle belt fusions showing large (row 5-like) denticles. (D and D') Wg loss-of-function caused a ventral lawn of denticles. Note that these are large denticles with a small refringent spot (row 5 denticles) resembling those seen in Mad RNAi depletion. (E) Dpp^H46^ mutant embryo showing fusion of two denticle belts. (F and F') Overexpression of UAST-MGM driven by mat-Gal4-VP16 caused patches of naked cuticle at the expense of denticle rows. (G–I) Embryos stained for Engrailed at stage 9, showing that Mad depletion disrupts abdominal segmental bands, while MGM overexpression does not.

The Mad pathway has not been explicitly implicated in the overall patterning mechanism of *Drosophila* segments previously, although some indications existed in the literature (see discussion below). *Drosophila* segmentation is known to be controlled by the Wg, EGFR and Hh pathways [25]–[27]. However, our phospho-specific antibodies suggested a possible regulatory role for Mad linker phosphorylations during segmentation (Figure 3J–3O). To investigate this further, we examined segmentation in embryonic cuticles. When Mad was maternally depleted, embryos displayed segmental patterning defects (Figure 6). The ventral denticle belts were expanded along the D–V axis (Figure 6A and 6B) and displayed fusions between segments (Figure 6C and C'). The denticle fusions were caused by lawns of denticles that replaced naked cuticle. Interestingly, the type of denticle induced by Mad RNAi was indistinguishable from the large denticles (row 5) observed in Wg null cuticles (compare Figure 6C'–6D'; [28]). Although to our knowledge denticle fusions have not been described in the literature in Dpp mutants, occasional denticle belt fusions could be observed in *dpp^H46^* nulls (Figure 6E).

In the converse experiment, overexpression of MGM, but not of MWT, caused the replacement of denticle belts by regions of naked cuticle (Figure 6F and 6F'). As seen in Figure 6F, this cuticle lacked dorsal hairs and therefore was true ventral naked cuticle and not the result of embryonic dorsalization caused by excessive BMP/Mad^Cter^ signaling. A similar naked cuticle phenotype is observed when Wg is overexpressed ([7] and data not shown).

The segmentation process in *Drosophila* occurs in multiple stages, beginning with the expression of gap, pair-rule and segment polarity genes [25]. Denticle rows are relatively late markers of this process. We therefore examined the expression of Engrailed, a gene that is regulated by both Wg and Hedgehog, at germ band extension stage. In maternally-depleted Mad RNAi embryos Engrailed stripes were patchy in abdominal segments (Figure 6G and 6H). In embryos overexpressing MGM, which develop naked cuticle, the engrailed stripes were relatively normal, but there was a slightly expanded engrailed expression in some anterior segments (Figure 6I) consistent with increased Wg activity. Thus, maternal Mad has an early role in the segmentation process.

Taken together, these results indicate that Mad is involved in segmental patterning in *Drosophila*, phenocopying loss- or gain-of-function of Wg signaling. Wg regulates the phosphorylation state of Mad during segmentation (as shown in Figure 3M and 3R), offering a possible node for integration of the Wg, EGFR/MAPK and BMP signaling pathways. Mad presumably works at the transcriptional level in combination with other Wg pathway intracellular components such as Armadillo/β-Catenin and Pangolin/Lef1 [29].

### Smad5/8 Is Required for *Xenopus* Segment Border Formation

**Figure 7:**
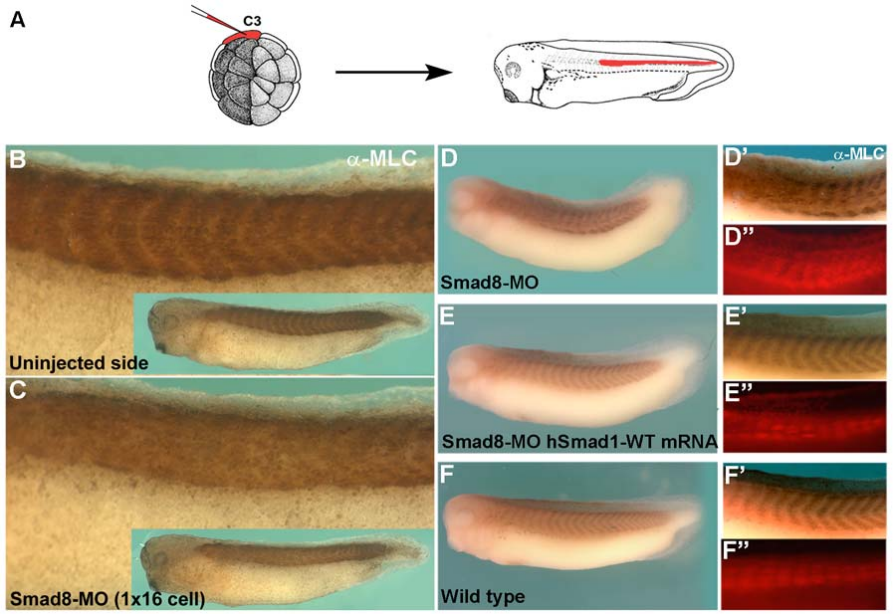
Smad5/8 Is Required for Segment Border Formation in *Xenopus* Embryos. (A) Illustration of C3 *Xenopus* blastomere injection at the 32 cell stage. The fate of C3 cells in the somites is indicated in a stage 28 tadpole. (B and C) In *Xenopus*, microinjection of Smad8-MO at the 16 or 32 cell stage (in C2 or C3 blastomeres) erased segmental somite borders on the injected side. Somites are composed mostly of segmental muscles, which were stained for myosin light chain (α-MLC). (D-F″) Smad8-MO effects on segment borders were cell autonomous (co-injection at 32-cell stage with rhodamine dextran amine lineage tracer), and were rescued by human *Smad1* mRNA (n = 55, 44 with somite fusions, n = 19, with 17 completely rescued, n = 24, all normal, respectively, 3 independent experiments).

Since the mechanisms of development have been conserved through evolution, we tested whether Smads are involved in segment formation in the vertebrates. In *Xenopus laevis*, the main maternally expressed Smad has been designated *Smad8* [30]. This gene probably corresponds to the closely related and maternally-expressed zebrafish Smad5 [10]. We developed an antisense morpholino oligo (MO) for *xSmad8*, which caused dorsalization (anti-BMP) phenotypes (Figure S11). Smad8-MO was injected into single blastomeres at the 16 or 32 cell stage in the region from which somites arise (C2 and C3 blastomeres), and embryos were stained for myosin light chain with 12/101 monoclonal antibody (Figure 7A). It was observed that muscle differentiation occurred normally, but on the injected side the segmental borders were erased (compare Figure 7B and 7C). Experiments using lineage tracer co-injection showed that somite border disruption was cell autonomous (Figure 7D–7D″). The segmentation phenotypes caused by Smad8 depletion were specific, as they were rescued by co-injection of human *Smad1* mRNA (Figure 7E–7E″). In addition, segmentation was also disrupted by microinjection of GSK3-resistant activated forms of Smad1 (Figure S12). These results lead us to the unexpected conclusion that the Mad/Smad5/8 transcription factor is required for segmentation both in *Drosophila* and *Xenopus*. The experiments do not allow us to conclude whether the same biochemical step is affected in both organisms, but this is of evolutionary interest that the Smad5/8/Mad transcription factor is now found to be required for segmentation in such diverse species

## Discussion

This study was initiated as an attempt to dissect the molecular mechanisms by which the Mad transcription factor integrates signals from three signaling pathways – Dpp, MAPK and Wg/GSK3 – using *Drosophila* as the readout. Emphasis was placed in demonstrating that Mad is required for Wg signaling. New reagents were generated, including phospho-specific antibodies for Mad GSK3 and MAPK phosphorylations, and Mad RNAi transgenic flies in which the maternal stockpile of *mad* mRNA can be partially depleted. Two main findings emerged. First, that Mad is required for Wg signaling in multiple in vivo assays. Second, that Mad was found to be phosphorylated in a segmental pattern and to be required for segmental patterning.

### Mad is Required for Wg Signaling

Transgenic flies expressing forms of Mad resistant to GSK3 phosphorylation displayed high BMP and Wg signaling phenotypes (Figures 1 and 2). Mad contains 74 serines/threonines, yet phosphorylation-resistant mutations of a single MAPK or of two GSK3 sites generated hyperactive transcription factors. Previous work in *Drosophila* had identified that phosphorylation by the Nemo/NLK kinase in the MH1 domain of Mad inhibits its activity [31], and that a neomorphic human Smad4 mutation can produce Wg-like phenotypes when overexpressed in the wing [32]. During *Drosophila* early embryogenesis, Mad linker phosphorylations tracked the priming activity of MAPK/EGFR, particularly in the ventral side, suggesting that Mad may be regulated independently of dorsal Dpp signals. *Drosophila* EGFR activates MAPK in a broad ventral region which corresponds to the neurogenic ectoderm [18]. Although this study focused on the role of Wg/GSK3 on Mad regulation, the priming phosphorylation for GSK3 is provided by EGFR signaling and is critical for Mad to be polyubquitinated and degraded in the centrosome.

Mad MGM, which mimics Mad receiving a maximal Wg signal, phenocopied known Wg overexpression phenotypes. In the wing, MGM caused the formation of ectopic rows of Senseless-expressing cells, sensory bristles, and entire ectopic wing margins (Figure 2). In larval cuticles, MGM caused the reduction of ventral denticle belts, which were replaced by naked cuticle regions, an indicator of Wg signaling. The *mad* gene product was demonstrated to be involved in Wg signaling in multiple in vivo assays. In the wing disc, Wg overexpression strongly increased *senseless*, *distalless*, *optomotor blind*, and *vestigial* transcripts, and co-expression of Mad RNAi inhibited this effect, without affecting Wg expression levels (Figure 4). The induction of ectopic neurogenic ectoderm tissue positive for SoxNeuro by RNAi was epistatic to the inhibition of SoxNeuro expression caused by Wg overexpression (Figure 5G–5J). Taken together, these results suggest that Mad is a required component for several Wg signaling events in *Drosophila*.

### Mad is Required for Segmental Patterning

Segmentation phenotypes were observed when Mad RNAi was expressed maternally using the pUASp vector [24]. Segment fusions were generated in which larval naked cuticle was replaced by large denticles of the same type (row 5) as those seen in Wg nulls [28]. In gain-of-function experiments, overexpression of GSK3-resistant Mad caused denticle belts to be replaced by naked cuticle, mimicking Wg signaling (Figure 5). Thus, depletion or overexpression of Mad generated Wg-like phenotypes, indicating that Mad functions in the Wg signaling pathway during segmental patterning.

The MAPK pathway, which during *Drosophila* embryonic segmentation is regulated by EGFR activity [26], [33], would decrease the duration of the Mad signal by promoting Mad polyubiquitination and degradation [4]–[5]. The EGFR-activating genes *rhomboid* and *spitz* are activated in the anterior of each segment and Wg in the posterior border of the anterior compartment [26], [33]. Wg/Wnt signals would increase the duration of the signal by inhibiting GSK3 phosphorylations (Figure 3M; [5]), generating a double gradient of GSK3 and MAPK activities that would regulate Mad stability and signaling within each segment. This may occur in a Dpp-independent fashion, but it is also possible that BMP signals might be active during larval segmentation, since the expression of the BMP receptor *thickveins* has a segmental pattern of expression [34]. In addition, Dpp is expressed in the ectoderm during segmentation stages, and its promoter contains segmentation modulation elements [35].

Finding a role for Mad in segmentation was remarkable, because this process has been extensively studied in *Drosophila* genetic screens [25], [13] and Mad had not been previously implicated as part of the segmentation machinery. This new role for Mad can be explained by the fact that Mad appears to also function independently of Dpp and that the Mad^10^ and Mad^12^ null alleles are nulls only for the BMP pathway. This persistence of a Mad linker regulation by phosphorylation could explain results in the literature showing that Mad^10^ mutant clones can result in Wg-like effects in the *Drosophila* wing [36]. Overexpression of *Mad^12^* synthetic mRNAs mutated in the GSK3 phosphorylation sites have strong posteriorizing activity in *Xenopus* embryos, as shown in Figure S7. This indicates that Mad mutants previously thought to be nulls retain BMP-independent functions.

As mentioned in the results, there were previous indications of a role for Dpp during segmentation in the *Drosophila* literature, and perhaps others exist of which we are unaware. Ferguson and Anderson [37] noted that in hypomorphic mutations of the BMP antagonist *short gastrulation* (*sog*, the homolog of Chordin), four copies of *Dpp* caused loss of some denticles and an increase in naked cuticle. In addition, Takaesu et al. [38] reported that in Dpp null mutants the posterior spiracles are replaced by an ectopic denticle belt. As noted here, Dpp nulls can present denticle belt fusions, a phenotype that has been observed previously in embryos injected with noggin mRNA [39]. Dpp null phenotypes and those of Mad^10^ and Mad^12^ mutants (which lose C- terminal, but not linker phosphorylations, Figure 4A–4E) are not identical to those of Mad RNAi. We suggest this is because Mad also has Dpp-independent functions. Dissecting which effects of Mad are Dpp-dependent and which ones are independent will be an interesting area of future investigation. In this study we present evidence for both taking place (e.g. Figure 3 and Figure 5A and 5B).

Future work will have to address the level at which Mad regulation by MAPK and GSK3 interacts with other intracellular components of the Wg transduction pathway that result in similar phenotypes. The phenotypes observed for Mad loss-of function and mad phosphorylation-resistant linker mutants overexpression were very similar to those found for the *armadillo/β-catenin*, *pangolin/lef1*, *legless/bcl9* and *pygopus* genes [29], [40]. The present study does not resolve the issue of whether the stabilized forms of Mad interact directly at the protein-protein binding level, thus modifying the core Wg pathway, or at level of DNA enhancers. Wnt responsive enhancers frequently contain Smad binding sites near TCF/Pangolin binding sites [38], [41], [42]. In the vertebrates, direct binding between Lef1/Tcf and Smads 1 to 4 at the level of enhancer binding sites has been known for some time [42]–[44]. What we now show here is that Mad is also directly regulated at the level of its phosphorylation at GSK3 sites by Wg signaling (Figure 3). The possibility that Wg-stabilized Mad may bind to Armadillo/β-catenin, Pangolin/lef1, Legless/bcl9 and Pygopus independently of nearby Mad binding sites cannot be excluded at present. Mechanistic studies will have to explain the remarkable similarities between the stabilized Mad phenotypes and those of canonical Wg phenotypes in wing discs and bristles, segments and in neurogenic ectoderm in *Drosophila*, which suggest a widespread requirement for Mad in Wg signaling. Another aspect that will need to be addressed is why in *Xenopus* Wnt signaling through Smad1 has a complete requirement for β-Catenin [5], and in *Drosophila* the MGM can induce senseless only in regions in which β-catenin is also stabilized (Figure 2).

### The Ancestry of Segmentation

Many developmental mechanisms have been conserved during evolution [11], but segmentation is one in which commonalities between *Drosophila* and the vertebrates have not been found. Segmentation in vertebrates relies on the cyclic oscillation of *Notch* pathway transcripts in the posterior paraxial mesoderm [45]. In theory, Smad1/5/8 could provide an attractive regulator of the segmentation clock, because BMP signals have a duration of 1–2 hours in cultured cells, which can be extended by inhibiting GSK3 [5]. Wnt pathway genes cycle rhythmically in vertebrates [45], offering an interesting possibility for regulating Smad5/8 activity. Notch is required for segmentation in spiders, but not in *Drosophila* [46]. Recently, it has been found that in the cockroach, an insect in which the segments are formed sequentially in a posterior growth zone (and not simultaneously as in *Drosophila*), stripes of *Delta* and *Hairy* mRNA (two genes of the Notch pathway) cycle rhythmically as in the vertebrates [47]. We have now found that Smad5/8 is required for the formation of segmental boundaries in *Xenopus* somites and that Mad is required for *Drosophila* segment patterning. However, the results do not establish whether similar molecular steps are affected in both organisms. The conservation of this unexpected conserved role for Mad/Smad is important from an Evo-Devo perspective because it suggests that the last common ancestor shared between *Drosophila* and vertebrates, Urbilateria, might have been segmented [48].

### Conclusions

These studies on *Drosophila* Mad have uncovered an unexpected role for Mad in the Wg signaling pathway. Mad/Smads are transcription factors that have low binding affinity for DNA and require other DNA binding proteins as co-factors in order to recognize the promoters and enhancers of hundreds of target genes [1]. Future work will have to address how Mad or its partner Medea/Smad4 interact with proteins such as Armadillo/β-Catenin and Pangolin/Lef1 on Wnt-responsive promoters in *Drosophila*. The present study shows that Mad is required for Wg to signal, through its GSK3 phosphorylation sites, in a number of different in vivo assays. These include wing margin formation, sensory bristle induction in the wing, induction of the Wg induced gene *senseless*, the repression of neurogenic ectoderm, and segmental patterning. We propose that Mad serves as an integrator of patterning signals, which determine embryonic positional information. The finding that three major signaling pathways – MAPK, Wnt/GSK3 and BMP – are integrated at the level of Mad/Smad1/5/8 both in *Drosophila* and in the vertebrates has interesting implications for the evolution of animal forms through variations on an ancestral gene tool-kit [11].

## Materials and Methods

### Drosophila Strains

Transgenes and mutant alleles used in this work were as follows:

Mad-flag wild type UAST transgene on chromosome 3 was *yw;Bl/Cyo;MWT/MWT*, and on chromosomes 2 and 3 *yw;MWT/MWT;MWT/MWT* with *Cyo* and *TM6B* floating. Mad MAPK Mutant was *yw;Bl/Cyo;MMM/MMM*. Mad GSK3 Mutant was *yw;MGM/MGM;TM2/TM6B* or *yw;MGM/MGM;MGM/MGM* with *Cyo* and *TM6B* floating. Mad RNAi (nucleotides 226-807 in pWiz) was *yw;MAD-RNAi/MAD-RNAi;TM2/TM6B* or *yw;Mad-RNAi/Mad-RNAi;Mad-RNAi/MadRNAi*. For maternal expression, pUASp driven by the p-element transposase promoter was used to generate *yw;Bl/Cyo;Mad-RNAi/Mad-RNAi*. For RNAi rescue experiments we used a UAS human Smad1 kindly provided by S. J. Newfeld to generate yw;Mad-RNAi/Mad-RNAi;hSmad1/hSmad1. For epistatic experiments a homozygous UAS-Wg on chromosome 2 was used (Bloomington #5918), as well as a double homozygote Mad-RNAi;Wg strain, yw; Mad-RNAi/Mad-RNAi;Wg/Wg with Tm6B floating. Primers and methods for quantitative RT-PCR, embryo preparations, and immunostaining are available in Supplemental Experimental Procedures.

### *Drosophila* Transgenic Constructs

Full-length N-terminal flag-tagged MWT was cloned into the *Xenopus* expression vector PCS2+ using BamHI and XbaI restriction sites. MMM was generated by mutating serine 212 into Alanine, and MGM by mutating serines 208 and 204 into alanines. Point mutations were made with the Stratagene Site Directed Mutagenesis kit. MWT, MMM and MGM were subcloned into the pUAST vector [15] using BglII and XbaI sites, and stable transgenic fly lines generated by microinjection. Two Mad RNAi *Drosophila* lines were generated. The one used throughout this paper targeted the N-terminal domain (Mad RNAi 5′nucleotides 226-807). The second, which gave weaker phenotypes, targeted the C-terminal domain (Mad RNAi 3′nucleotides 657-1230). PCR fragments cloned into pGEMT-easy (Promega) were digested and inserted in opposite orientations in pWiz RNAi vector on either side of a *white* intron spacer [19], and transgenic lines made by Bestgene (Chino Hills, CA.). Because UAST is not expressed in the oocyte, maternal expression of Mad RNAi 5′ was achieved by excising the pWiz insert with Not1 and Xba1 and subcloning it into the pUASp vector [24].

### *Drosophila* Gal4 drivers

Gal4 drivers used (Bloomington stock number in parentheses) were as follows: *Actin5c-Gal4* (gift from J. Merriam), *Apterous-Gal4* (gift from M. Affolter), *Daughterless-Gal4* (#5460), *Dpp-Gal4* (gift from K. Pappu), *Mat-Gal4VP16* (7063), *MS1096-Gal4* (#8696), *Paired-Gal4* (#1947), *Patched-Gal4* (gift from K. Pappu), *Scalloped-Gal4* (#8609), vestigal-gal4 (#8222) and A9-gal4. Other strains used in this study were: *Brinker-LacZ* [20], *UAS-Wg* (#5919), *Dpp* null (#2061), *wg* null (#2980). The human UAS-Smad1 fly used for Mad RNAi rescue was described by Marquez et al. [48].

### Clonal Analysis

For random “flp-out” clones [17] we crossed females of the genotype y w;Act>y^+^>Gal4;UAS-GFP (kind gift of K. Pappu) to the following males: ywhsflp; MWT/MWT, ywhsflp;MGM/MGM or ywhsflp;Mad-RNAi/Mad-RNAi, all Mad transgenes on chromosome 2. Flies laid eggs for 8 hr, which were incubated for a further 16-20 hr. Larvae at the first instar were administered single heat shocks (32.5–37°C) ranging from 5–30 min for Mad RNAi and 20–60 min for MWT or MGM. After heat-shock, larvae were grown at room temperature for recovery and further development.

### Phospho-specific Antibodies

Antibody reagents specific for *Drosophila* phospho-Mad^MAPK^ (p-serine 212) and phospho-Mad^GSK3^ (p-serine 208) were generated. Two synthetic peptides (NSNPNS[PO3]PYDSLAGT) for the pMAD^MAPK^ and (SPSSVNS[PO3]NPNSPY) for the pMad^GSK3^ proved to be highly antigenic (Covance Research Products). For immunostaining experiments crude antisera, at 1∶500 and 1∶250 dilutions, respectively, were used.

### Embryo Fixation and Immunostaining

*Drosophila* embryos were collected at the desired stage, dechorionated in 50% bleach and rinsed thoroughly using distilled H_2_O. Embryos were transferred to a glass scintillation vial containing 50% heptane, 50% PEMFA (PEM and 4% formaldehyde) solution and gently rocked between 10 and 20 mins. The lower PEMFA layer was removed and an equal volume of methanol was added to the remaining heptane solution. The vial was then vigorously shaken for 30 seconds and the embryos were allowed to settle to the bottom. The methanol/heptane solution was removed and embryos were washed 3 times with 100% methanol. Fixed embryos can be stored at this point in 100% methanol at −20°C for several months. Embryos were stepwise rehydrated in 0.2–0.5% Triton X-100 in PBS and incubated for 1–2 hours with gentle rocking. Embryos were then incubated for 1 min in 0.5% SDS and rinsed in PBS/0.2% Triton X-100 for 5 min, followed by 1 hour incubation in blocking solution (PBS/20% goat serum, 2.5% BSA). The SDS treatment serves to make the antigen more accessible. For whole-mount embryo immunostaining, the primary antibodies used were rabbit α-pMad^MAPK^ (1∶500, crude antiserum), α-pMad^GSK3^ (1∶250, crude antiserum), α-Flag (1∶500, Sigma) and monoclonal antibodies used were α-Wg (1∶200), α-BP102 (1∶8), α-SoxN (1∶1000, gift of M. Bueschar) and γ-Tubulin (1∶500, Sigma), which were incubated overnight in blocking solution at 4°C. Embryos were washed 10 times for 10 min each using PBS/0.2% Triton X-100 before applying secondary anti-rabbit Alexa-488 conjugated antibodies (1;1000, Molecular probes) and anti-mouse Cy3-conjugated antibodies (1∶1000, Jackson Labs) for 1 hour at room temperature. After washing 10 times with PBS/0.2% Triton X-100, *Drosophila* embryos were mounted on glass slides using DAPI-containing Vectashield (Vector).

### *Drosophila* Embryo Chitinase Treatment

For pMAD^GSK3^ staining at late stages of development fixed embryos were treated with Chitinase. After embryos were rehydrated, 3 µg/ml of Chitinase in PBS (C6137 Sigma-Aldrich) was added and embryos incubated at room temperature overnight with gentle rocking. Embryos were then washed four times in PBS for one hour, followed by eight washes in PBS/0.2% Triton X-100 for 2 hours.

### Wing Disc Fixation and Immunostaining

Wing discs were dissected out of third instar larva in cold 0.02% Triton X-100 PBS (PBST) solution. Discs were fixed in 4% formaldehyde for 20 minutes on ice and rinsed using PBST. Discs were then incubated in blocking solution (2.5% BSA and 5% goat serum in PBS/0.02% Triton X-100) for 1–2 hours at room temperature. Primary antibodies α-Senseless (1∶10), α-Armadillo (1∶10) or α-Wg (1∶200) were incubated in blocking solution overnight at 4°C and washed 10 times for 2 hours in PBST. Discs were incubated for 1–2 hours in blocking solution and incubated for 1 hour in anti-mouse Cy3-conjugated secondary antibody (1∶1000, Jackson Labs) at room temperature. Wing discs were placed in DAPI-containing Vectashield (Vector) overnight and mounted on glass slides.

### Microscopy

Fluorescent images were photographed with a Zeiss Axiophot or an Axio Imager.Z1 microscope. The Axio Imager.Z1 microscope was equipped with Zeiss ApoTome oscillating grating in the epifluorescence beam, which significantly reduces out of focus stray light.

### Cuticle Preparations

We followed in general the methods described by Wieschaus and Nusslein-Volhard [50]. Larvae were collected 24 hrs after egg laying, dechorionated in 50% bleach for 2 mins, rinsed in distilled H_2_O and placed into a glass scintillation vial containing 50% methanol and 50% heptane. The glass vial was then shaken for 30 seconds. The upper phase of heptane was removed and larvae washed with 100% methanol three times. Methanol was removed and embryos transferred into acetic acid/glycerol (3∶1) solution, and incubated for at least one hour at 70°C. After incubation, the acidic acid/glycerol mix was removed completely, and 150 µl of mounting medium (Hoyer's medium) was added to the larvae where they were left to soak for 15–30 mins. The larvae were carefully dropped onto a glass slide and a coverslip was placed over. 10 g weights were placed on the coverslip to flatten the cuticles for one day in a 70°C oven. Cuticle preps were visualized using dark field microscopy.

### Mounting of Adult Wings

Wings were removed from adult flies and dehydrated in 100% ethanol for 5 mins. The wings were placed onto a slide with the dorsal side up, and the ethanol was allowed to evaporate. A small drop of Canada balsam was dropped onto the wing and a glass coverslip was placed on top. A 10 g weight was used to flatten the preparation.

### RNA Isolation and Quantitative RT-PCR

For wing disc samples, total RNA from ten wing discs from third instar larvae were extracted using the Absolutely RNA Microprep Kit (Stratagene). cDNA synthesis was carried out using random hexamer priming and the StrataScript Reverse Transcriptase. For whole embryo samples, embryos on agar plates were covered with Halocarbon 700 oil 4–6 hrs after egg laying. This treatment makes the embryos transparent (after approximately 10 min in oil) and allows one to distinguish live and dead embryos. 50 embryos per sample were picked from grape plates, transferred into lysis buffer, supplied by the Absolutely RNA Miniprep Kit (Stratagene), and immediately frozen on dry ice to break the chorion. For extraction of total RNA, the samples were thawed and homogenized in 0.1 ml ground glass homogenizers until no intact embryos were visible, before proceeding with cDNA synthesis. Quantitative RT-PCR was performed using the Mx3000P machine (Stratagene), and the Brilliant SYBR Green QPCR Master Mix (Stratagene). Three independent batches of wing discs or whole embryos were analyzed. Measurements were performed in quadruplicates and normalized to the expression levels of *Rp49* (*RpL32*, *Ribosomal protein L32*) or *Gal4*. Fold change values (*x*) were calculated using the following formula: *x* = 2^−ΔΔCt^. For calculation of relative transcript numbers per wing disc, standard curves of the control samples were measured. The primer sequences we designed were:

*Distalless* fwd: CTCCTACTCCGGCTACCATC, rev: ACCAGATTTTCACCTGCGTTT; *Gal4 (S.cerevisiae)* fwd: GGATGCTCTTCATGGATTTG, rev: CAACATCATTAGCGTCGGTGAG; *Hedgehog* fwd: GAGATGGAATCCTGGAAGAGC, rev: GTGGGTTTTTGATTTGTGGTG; *Optomotor blind* fwd: ACGGACTGGAGGTTCAACA, rev: ATGGTGCGAGTGTAGATGG; *Rp49* fwd: TACAGGCCCAAGATCGTGAA, rev: TCTCCTTGCGCTTCTTGGA [51]; *Senseless* fwd: CCGAAAAGGAGCATGAACTC, rev: CGCTGTTGCTGTGGTGTACT; *Spalt* fwd: CAAGGAGGATTTGGAGGATTC, rev: TCCGTAACCAGGCTGATATTG; *Vestigial* fwd: CCAGGGACAGGCTCAATATCT, rev: TGCCATACAAGTCGCTAACCT; *Wingless* fwd: GATTATTCCGCAGTCTGGTC, rev: CTATTATGCTTGCGTCCCTG. The PCR cycling conditions for 40 cycles were: denaturation at 95°C for 30 seconds, annealing at 55°C for 60 seconds, and extension at 72°C for 30 seconds.

### Morpholino, mRNA Injection and Whole-mount in *Xenopus* Situ Hybridization

The antisense morpholino (MO) for *X. laevis Smad8* (5′-TGCATTGGATTTGCTGTGTTTACC-3′) was purchased from Gene Tools LLC. The Smad8-MO (0.5 mM) was initially injected four times radially into *Xenopus* embryos to test for low BMP phenotypes. For segmentation experiments, all injections were into a single C-tier (C2 or C3) blastomere at the 16 or 32 cell stage (the region from which somites arise). mRNAs or MOs were coinjected with either 10 pg nuclear lacZ DNA or 0.5% rhodamine dextran amine lineage tracer. In knockdown experiments, embryos were injected with Smad8-MO (4 nl, 0.3 mM) alone or in combination with *Smad1-WT* mRNA (100 pg) to rescue somite borders. In overexpression experiments total amounts of Smad1 mRNAs (*Smad1-WT*, *Smad1-GM*, *Smad1-SEVE* and *Smad1-SEVE-GM*, [5]) injected per blastomere was 100 pg. The procedures for mRNA synthesis and *Xenopus* whole-mount in situ hybridization are available at www.hhmi.ucla.edu/derobertis/index.html.

### LacZ Lineage Tracing and Whole-mount Immunostaining

For lacZ lineage tracing, *Xenopus* embryos were fixed for 20 min. in MEMFA [52], and washed twice in PBS for 10 min. β-Gal staining was performed in 0.5 M K_3_Fe(CN)_6_, 0.5 M K_4_Fe(CN)_6_, 0.5 M MgCl_2_ and 100 mg/ml 5-Bromo-6-chloro-3-indolyl-β-D-galactopyranoside (in DMSO) in PBS at 4°C overnight.

For muscle staining, embryos were washed twice in PBS and re-fixed in MEMFA for 2 hours [53], followed by washing in PBST for 1 hour. Embryos were then incubated in blocking solution (PBS-Tween (PBST: 0.1% Tween-20 in PBS, 5% BSA, 5% goat serum) for 1 hour at room temperature and then incubated in blocking buffer for 24–48 hrs at 4°C with a myosin light chain antibody 12/101 (1∶10 dilution, obtained from Developmental Studies Hybridoma Bank, University of Iowa, [54]). Embryos were washed 10 times in PBST (2 hrs total) at room temperature and blocked at room temperature for a further 1 hour. Embryos were incubated in secondary antibody (α-mouse IgG-HRP 1/250, Amersham) overnight at 4°C, and washed 10 times in PBST. For HRP staining, the DAB-solution (Roche) was prepared and NiCl_2_ was added to a final concentration of 1% to enhance the staining. The color reaction was monitored until the brown signal appeared (approx. 5 min). The embryos were washed in PBST to stop the staining reaction.

### Western blot of *Drosophila* embryos and S2 cells

Fifty wild type or UAST Mad-RNAi (under the control of Daughterless-Gal4) *Drosophila* embryos were collected at stage 15. After homogenization using 150 µl of lysis buffer in a Pyrex ground glass homogenizer, extracts were analyzed by Western blot. For analysis of *Drosophila* S2 cells, 1–1.5×10^6^ of cells were transfected with 0.3 µg of total DNA (0.05 µg of pUAST-Mad, 0.05 µg of Gal4 and 0.2 µg of pUAST-Mad-RNAi or pUAST empty vector DNAs) using Effectene Transfection Reagent. One day after transfection, Gal4 protein expression was induced by the addition of CuSO_4_ (0.7 mM, [55]). 48 hrs later, cells were extracted using lysis buffer (50 mM Tris pH 7.4, 150 mM NaCl, 1 mM EDTA and 1% Triton X-100) and analyzed by Western blot. Primary antibodies used were: pMad^MAPK^ (1∶1500, [6]); pMad^GSK3^ (1∶1000, this study); pMad^Cter^ (1∶4000, [56]); Flag (1∶2000, Sigma); and Total Erk (1∶1000, Cell Signaling).

### Ubiquitination assay

293T cells were cotransfected with 2 µg of a 10∶2∶1 mixture of Mad-flag, ubiquitin-HA and *Drosophila* Smurf DNA into 6-well culture plate using Fugene6 transfection reagent (Roche). After 24 hrs cells were lysed in 200 µl lysis buffer (20 mM Tris HCl pH 8.0, 0.1% NP-40, 1 mM EDTA, 10% glycerol, 1× phosphatase inhibitor cocktail I and II (Calbiochem) and 1× protease inhibitor cocktail (Complete EDTA-free, Roche). Plates were rocked on ice for 15 min, scraped and transferred to 1.5 ml Non Stick Surface tubes (VWR). Lysates were cleared by centrifuging for 10 min at 4°C, the supernatant transferred to a fresh tube, 30 µl of anti-Flag M2 affinity gel (Sigma) added, and incubated at 4°C for 2 hr with end-over-end rotation. The resin was washed four times with lysis buffer, and eluted with 60 µl of 2× SDS-PAGE sample buffer, and run in a 4–15% precast gradient gel (Bio-Rad).

## Supporting Information

Figure S1 UAS-Mad Transgenes Are Expressed at Comparable Levels in Drosophila Embryos. (A) Western blot analysis of total Mad-Flag protein from individual first instar larvae expressing either Mad Wild Type (MWT), Mad MAPK Mutant (MMM) or Mad GSK3 Mutant (MGM) driven by act5c-Gal4. The Mad transgenes were detected using rabbit anti-Flag antibody. (B–D) Paired-Gal4 was used to drive UAS-MWT, MMM or MGM in Drosophila embryos and stained for anti-Flag. Note that similar levels of total MWT, MMM and MGM were seen by western blotting and whole-mount immunostaining, indicating that each UAS MAD transgene was driven at comparable levels.
(8.19 MB TIF)

Figure S2 Overexpression of Mad Phosphorylation-Resistant Mutants causes Ectopic Bristles. A) Wild-type anterior wing margin. (B) MMM (one copy) induced ectopic chemosensory bristles on the dorsal side of longitudinal veins one and two (arrowheads) when driven with sd-Gal4. (C) Overexpression of MGM (two copies) increased stout and chemosensory bristles on vein 1 (same panel as in Figure 2C) (D) Wild-type posterior wing margin. (E) Ectopic sensory bristles (arrowheads) are apparent up to 8 cell diameters away from the posterior wing margin when one copy of MMM is driven with Sd-Gal4 or A9-Gal4. Note that these bristles form directly on the wing blade, independently of vein formation (high BMP phenotype). (F) Cluster of ectopic bristles on the wing blade (arrowheads); these Wg-like phenotypes were caused by the two copies of MGM driven by A9-Gal4. (G) Overexpression of MMM (one copy) using A9-Gal4 induced ectopic chemosensory bristles (arrows and hatched box). (H) High magnification of ectopic chemosensory bristles on longitudinal vein 5 close to the wing hinge. Thus, MMM also causes Wg phenotypes, indicating that both MAPK and GSK3 phosphorylations play an important role promoting wing bristle formation.
(0.51 MB TIF)

Figure S3 Ectopic margin bristles are not induced by Dpp overexpression. (A) Wild type adult wing. (B) Overexpression of Dpp along the presumptive wing margin in larval wing discs fails to induce ectopic bristles in the adult wing. The overexpression was effective, because ectopic veins were formed due to Dpp overexpression close to the margin (arrowheads). Note also the wing is enlarged due to increased BMP signaling. Dpp was driven in the wing margin by the vestigal margin enhancer-gal4. This experiment shows that the bristles seen when MGM and MMM are overexpressed (Figures 2 and S2) are not caused by a change in normal BMP signaling through C-terminal phosphorylation.
(2.13 MB TIF)

Figure S4 Overexpression of MGM increases expression of Senseless, a Wg target gene in the wing margin. (A) Expression of Senseless marks future sensory cells in wing discs. In the prospective wing blade, two rows of Senseless positive cells flank the Wg-expressing stripe that demarcates the margin. These cells will later become the sensory bristles of the wing margin Inset shows magnification of anterior wing margin senseless-expressing cells. (B) Overexpression of MGM, driven by scalloped-Gal4 in the wing pouch, increased the number of cells expressing senseless protein (inset) and the overall size of the wing pouch. We note that senseless overexpression is higher in the anterior wing margin and tis strongest close to the Dpp expression domain.
(2.55 MB TIF)

Figure S5 Clonal Analyses of Overexpressed Mad Proteins in Wing Discs. (A and B) Clonal expression of MWT (marked by GFP) does not increase Senseless expression along the presumptive wing margin. (C–D) MWT or MGM flp-out clones in the anterior compartment of wing discs do not cause ectopic expression of Engrailed protein (which is expressed only in the posterior compartment). These results also indicate that Hedgehog is not expressed in the anterior compartment within or around these clones, since ectopic En would reprogram cells in the anterior compartment to express Hedgehog [57].
(4.58 MB TIF)

Figure S6 Asymmetric Immunostaining of pMadMAPK in Drosophila Cellular Blastoderm Cells. (A and B) Nuclear pMadMAPK visualized along a dorsal stripe. The nuclear pMadMAPK staining tracks pMadCter, which is dependent on Dpp signaling. A single bright cytoplasmic spot is apparent in most cells in stage 6 Drosophila embryos, which is seen in both nuclear and non-nuclear stained cells. (C–F) High power of a field of blastoderm cells, showing that the pMadMAPK spot is either adjacent or co-localizes with one of the centrosomes marked by γ-Tubulin. These cytoplasmic spots most likely represent Mad targeted for degradation to pericentrosomal proteasomes [6].
(6.17 MB TIF)

Figure S7 The Mad12 mutant, which lacks the C-terminal phosphorylation sites, retains posteriorizing activity in Xenopus, in particular when the GSK3 sites are also mutated. (A) Whole mount in situ hybridization of tail bud stage Xenopus embryos (n = 16). Embryos are stained for Rx2a (eye), Krox20 (Hindbrain) and Sizzled (Ventral/Belly). (B) Microinjection of Mad12 mRNA reduced the anterior head region of the embryo, indicated by decreased Rx2a expression (n = 15). (C) Elimination of the anterior head structures in MGM12 microinjected mRNAs (mutation of the GSK3 phosphorylation sites mimics Mad receiving a maximal Wg signal) resulted in a severely posteriorized embryo with almost complete loss-of Rx2a expression (n = 13). (D) Similar posteriorized phenotypes are generated when Wnt10b DNA is microinjected into Xenopus embryos. In addition to this, there is an increase in the expression of the BMP responsive gene sizzled, presumably because it affects the stability of endogenous Smad1/5/8 (n = 7).
(1.34 MB TIF)

Figure S8 Expression of Mad RNAi in S2 Cells Specifically Inhibits Mad. Flag-tagged Mad was transiently co-transfected with empty vector (minus lane) or a construct expressing UAST Mad RNAi in Drosophila S2 cells co-transfected with a Gal4 plasmid (plus lane). Note that the different phosphorylated forms of Mad (pMadCter, pMadMAPK and pMADGSK3), as well as total Mad (α-Flag), were significantly reduced by co-expression of Mad RNAi. The expression of total Erk serves as control for equal loading.
(2.86 MB TIF)

Figure S9 Mad RNAi Can Completely Block Wing and Haltere Development. (A) Loss of adult wings and halteres when two copies of UAS-Mad RNAi were driven with Scalloped-Gal4 at room temperature. Arrowhead indicates the location of the haltere in the wild-type fly. (B and C) Loss of wing margin accompanied by notching of the wing blade was found occasionally when UASp Mad RNAi (or UAST MAD RNAi, data not shown) was driven by scalloped-Gal4. These wing notches resemble a Wg loss-of function phenotype. Similar losses of margin tissue have previously been observed in weak genetic mutants in the Dpp/Mad pathway [32].
(6.07 MB TIF)

Figure S10 Mad RNAi does not Affect Hedgehog Expression. (A–C) Driving Mad RNAi or MGM with apterous-Gal4 in the dorsal compartment of the does not cause ectopic induction of a hedgehog-lacZ reporter in the anterior wing compartment. (D) Hedgehog mRNA levels are unaltered when Mad RNAi or Wg were overexpressed in the wing pouch using scalloped-Gal4. Expression also remained unchanged in wing discs when both Mad RNAi and Wg were co-expressed. (E) Dpp expression is increased when Mad is depleted in wing disc using scalloped-Gal4. However, this transcriptional increase in Dpp expression does not increase signaling because of the knockdown of Mad. Because Mad is depleted, these wings still display a Dpp loss-of function phenotype. Wg only fractionally decreased Dpp expression. These experiments serve as controls for the epistatic studies in Figure 4N–4Q, showing that the increase in marker genes caused by Wg, or the decrease caused by Mad RNAi, can not be explained by changes in Hedgehog or Dpp levels.
(8.72 MB TIF)

Figure S11 Knockdown of xSmad8 Dorsalizes Xenopus Embryos. (A) Whole-mount in situ hybridization for the pan-neural marker Sox2 in an uninjected control embryo, stage 22, dorsal view. (B) Injection of xSmad8 morpholino (0.5 mM, 4 nl injected four times radially) leads to expansion of the neural plate. (C) Control embryo stained for Otx2 (forebrain and midbrain marker) and Krox20 (hindbrain, rhombomeres 3 and 5), lateral view. (D) Smad8-depleted embryos are dorsalized (anti-BMP phenotype) and show expansion of head structures. It should be mentioned here that the original depletion of Xenopus laevis Smad8 by Miyanaga et al [30] yielded a very different result, namely apoptosis via activation of caspases. However, it should be noted that their methods for depletion were different. They used DNA oligonucleotides to deplete Smad8 transcripts in oocytes that were then subjected to maternal transfer and fertilization. We used morpholino oligos (of a different sequence) injected at the 4-cell stage, and therefore the depletion of maternal transcripts must have been less extensive. This explains why we did not observe apoptosis, but instead dorsalization (anti-BMP phenotype) of the embryo. The morpholino described here provides a useful reagent for knockdown of the maternal Xenopus laevis BMP-Smad. Smad8 probably corresponds to the homolog of zebrafish Smad5 [10] and is therefore referred to below as Smad5/8.
(6.06 MB TIF)

Figure S12 GSK3-resistant Activated Forms of Smad1 Disrupt Segmentation in Xenopus Embryos. (A and B) Immunostainings for myosin light chain showing loss of segmental borders in somites in the injected side (B) of a Xenopus embryo (stage 26), compared to the uninjected side. C2 or C3 blastomeres were injected with 100 pg of hSmad1 resistant to GSK3 phosphorylation [5] and 50 pg of nuclear LacZ mRNA (n = 32/42). (C–F) In situ hybridizations for the somite marker MyoD shows a disruption of the segmental pattern in Xenopus embryos (stage 30) injected with activated forms of Smad1. Uninjected control embryos express MyoD in the somitic segments in a typical chevron shape (n = 27). Overexpression of Smad1 wild-type mRNA (SWT) does not change this pattern (n = 21). An activated mutant of Smad1 that has phospho-mimetic amino acid substitutions on the C-terminus (the two most c-terminal serines mutated into glutamic acids, designated “SEVE” mutant [5]) displays mild disruptions of the somite pattern (n = 9/12), while the same phospho-mimetic form of Smad1 with an additional mutation in the GSK3-phosphorylation site in the linker (named “SEVE-GM”) exhibits strongly impaired segmentation (n = 14/18). Nuclear LacZ marks the injected cells.
(3.89 MB TIF)
