# Supplementary figures and images for: Mad Is Required for Wingless Signaling in Wing Development and Segment Patterning in Drosophila
Source: PLoS One. 2009 Aug 6;4(8):e6543. doi: 10.1371/journal.pone.0006543 (PMC2717371; doi:10.1371/journal.pone.0006543)

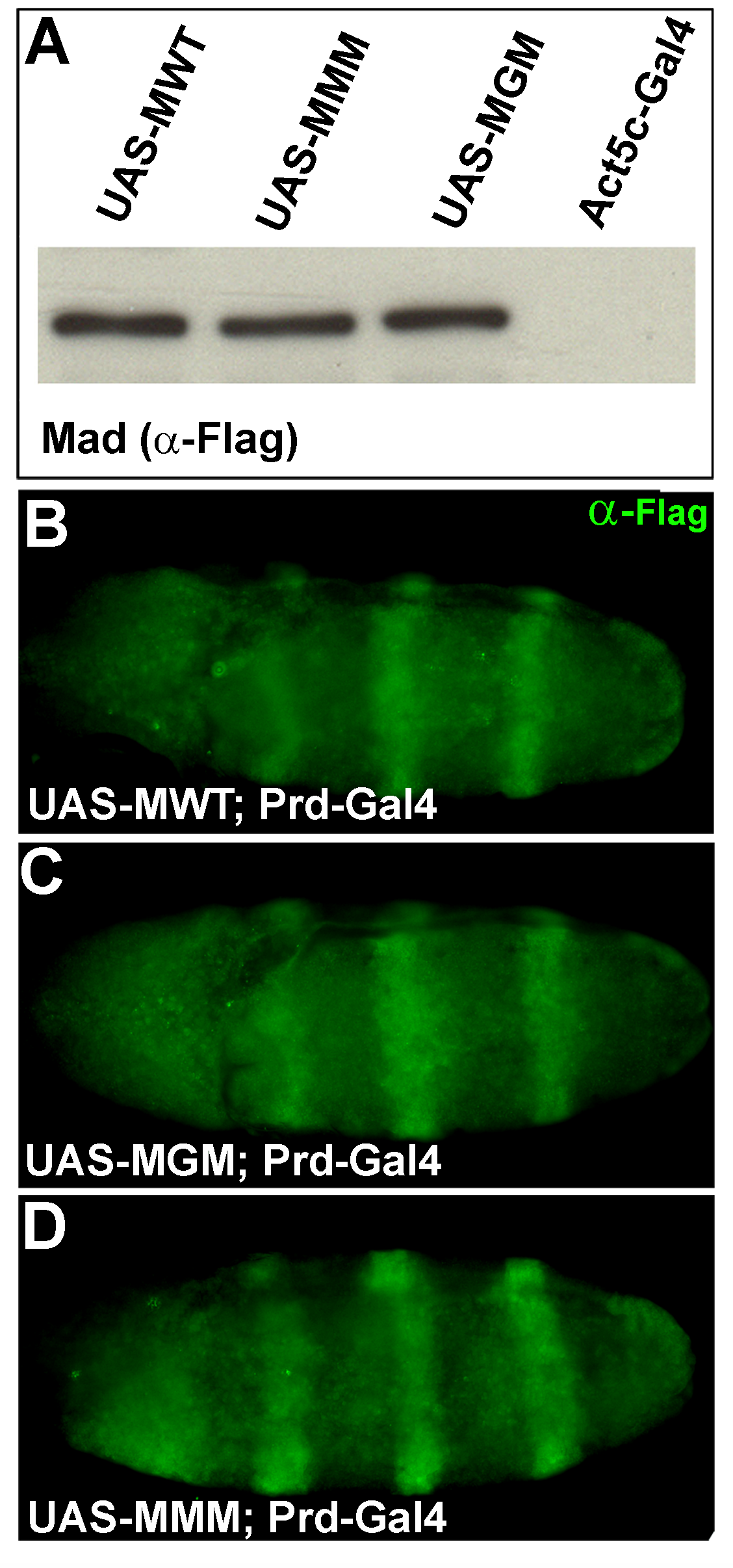

Supplement: Figure S1 — UAS-Mad Transgenes Are Expressed at Comparable Levels in Drosophila Embryos. (A) Western blot analysis of total Mad-Flag protein from individual first instar larvae expressing either Mad Wild Type (MWT), Mad MAPK Mutant (MMM) or Mad GSK3 Mutant (MGM) driven by act5c-Gal4. The Mad transgenes were detected using rabbit anti-Flag antibody. (B–D) Paired-Gal4 was used to drive UAS-MWT, MMM or MGM in Drosophila embryos and stained for anti-Flag. Note that similar levels of total MWT, MMM and MGM were seen by western blotting and whole-mount immunostaining, indicating that each UAS MAD transgene was driven at comparable levels. (8.19 MB TIF) [file pone.0006543.s001.tif]

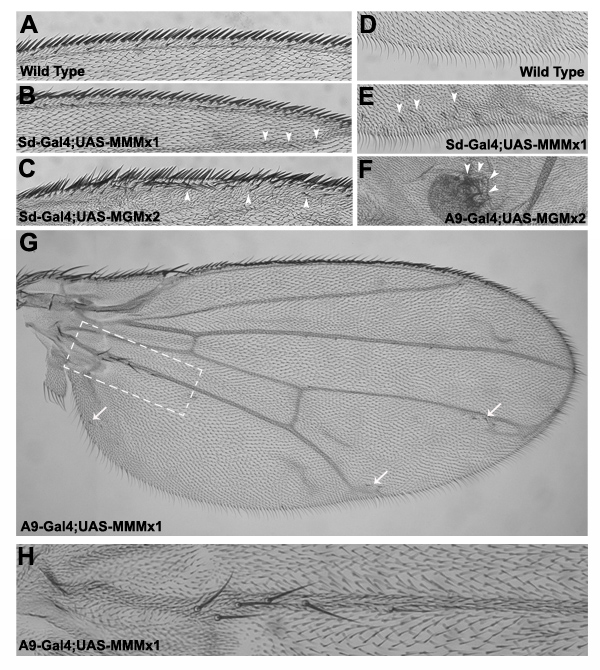

Supplement: Figure S2 — Overexpression of Mad Phosphorylation-Resistant Mutants causes Ectopic Bristles. A) Wild-type anterior wing margin. (B) MMM (one copy) induced ectopic chemosensory bristles on the dorsal side of longitudinal veins one and two (arrowheads) when driven with sd-Gal4. (C) Overexpression of MGM (two copies) increased stout and chemosensory bristles on vein 1 (same panel as in Figure 2C) (D) Wild-type posterior wing margin. (E) Ectopic sensory bristles (arrowheads) are apparent up to 8 cell diameters away from the posterior wing margin when one copy of MMM is driven with Sd-Gal4 or A9-Gal4. Note that these bristles form directly on the wing blade, independently of vein formation (high BMP phenotype). (F) Cluster of ectopic bristles on the wing blade (arrowheads); these Wg-like phenotypes were caused by the two copies of MGM driven by A9-Gal4. (G) Overexpression of MMM (one copy) using A9-Gal4 induced ectopic chemosensory bristles (arrows and hatched box). (H) High magnification of ectopic chemosensory bristles on longitudinal vein 5 close to the wing hinge. Thus, MMM also causes Wg phenotypes, indicating that both MAPK and GSK3 phosphorylations play an important role promoting wing bristle formation. (0.51 MB TIF) [file pone.0006543.s002.tif]

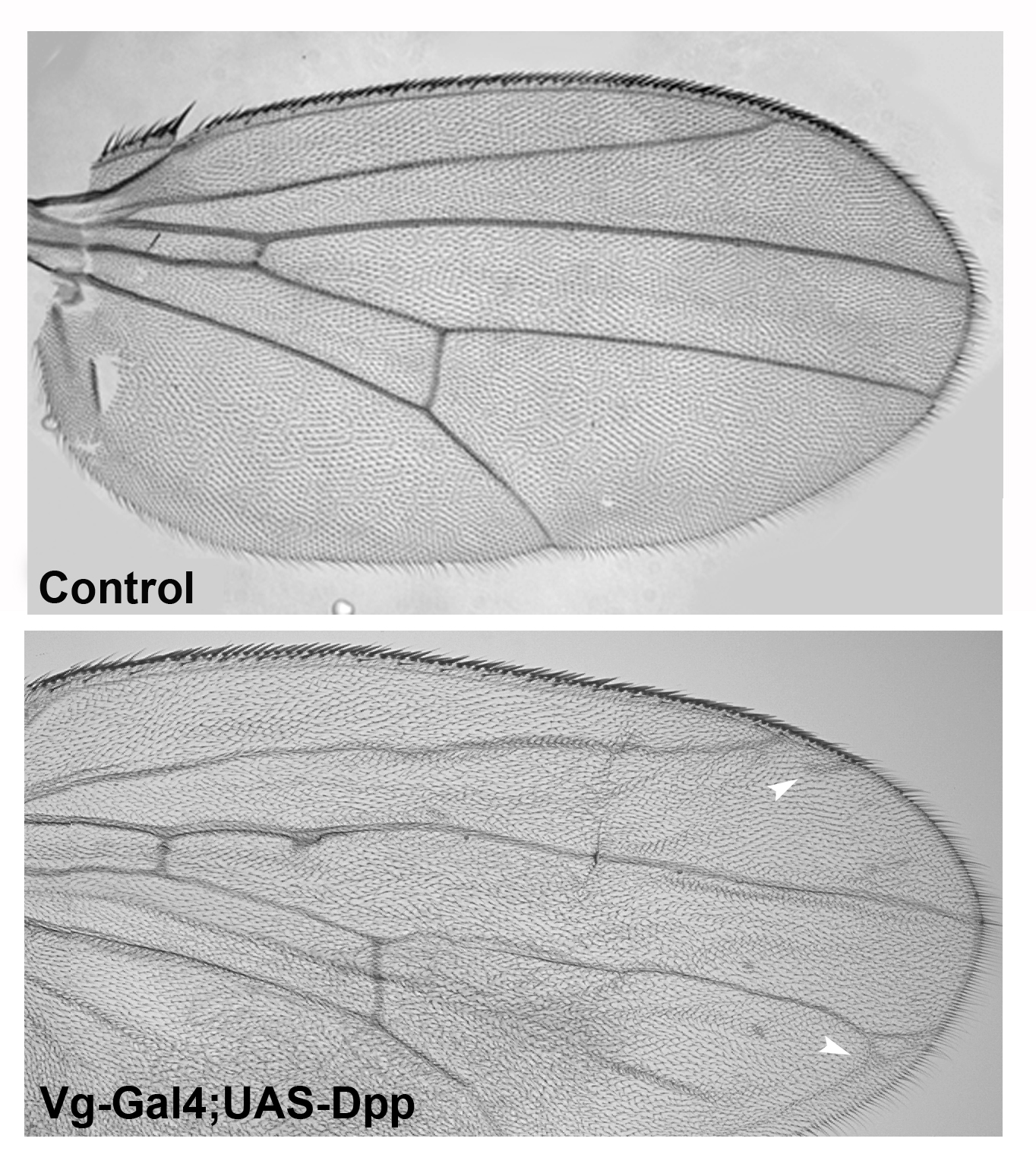

Supplement: Figure S3 — Ectopic margin bristles are not induced by Dpp overexpression. (A) Wild type adult wing. (B) Overexpression of Dpp along the presumptive wing margin in larval wing discs fails to induce ectopic bristles in the adult wing. The overexpression was effective, because ectopic veins were formed due to Dpp overexpression close to the margin (arrowheads). Note also the wing is enlarged due to increased BMP signaling. Dpp was driven in the wing margin by the vestigal margin enhancer-gal4. This experiment shows that the bristles seen when MGM and MMM are overexpressed (Figures 2 and S2) are not caused by a change in normal BMP signaling through C-terminal phosphorylation. (2.13 MB TIF) [file pone.0006543.s003.tif]

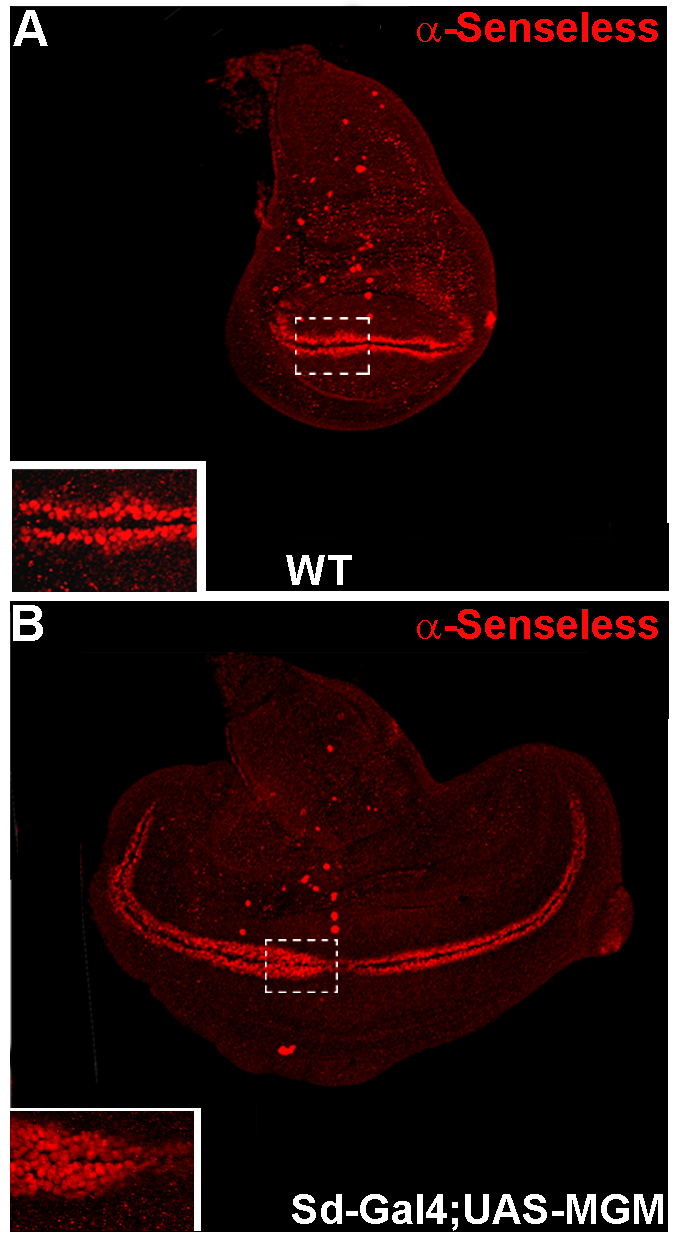

Supplement: Figure S4 — Overexpression of MGM increases expression of Senseless, a Wg target gene in the wing margin. (A) Expression of Senseless marks future sensory cells in wing discs. In the prospective wing blade, two rows of Senseless positive cells flank the Wg-expressing stripe that demarcates the margin. These cells will later become the sensory bristles of the wing margin Inset shows magnification of anterior wing margin senseless-expressing cells. (B) Overexpression of MGM, driven by scalloped-Gal4 in the wing pouch, increased the number of cells expressing senseless protein (inset) and the overall size of the wing pouch. We note that senseless overexpression is higher in the anterior wing margin and tis strongest close to the Dpp expression domain. (2.55 MB TIF) [file pone.0006543.s004.tif]

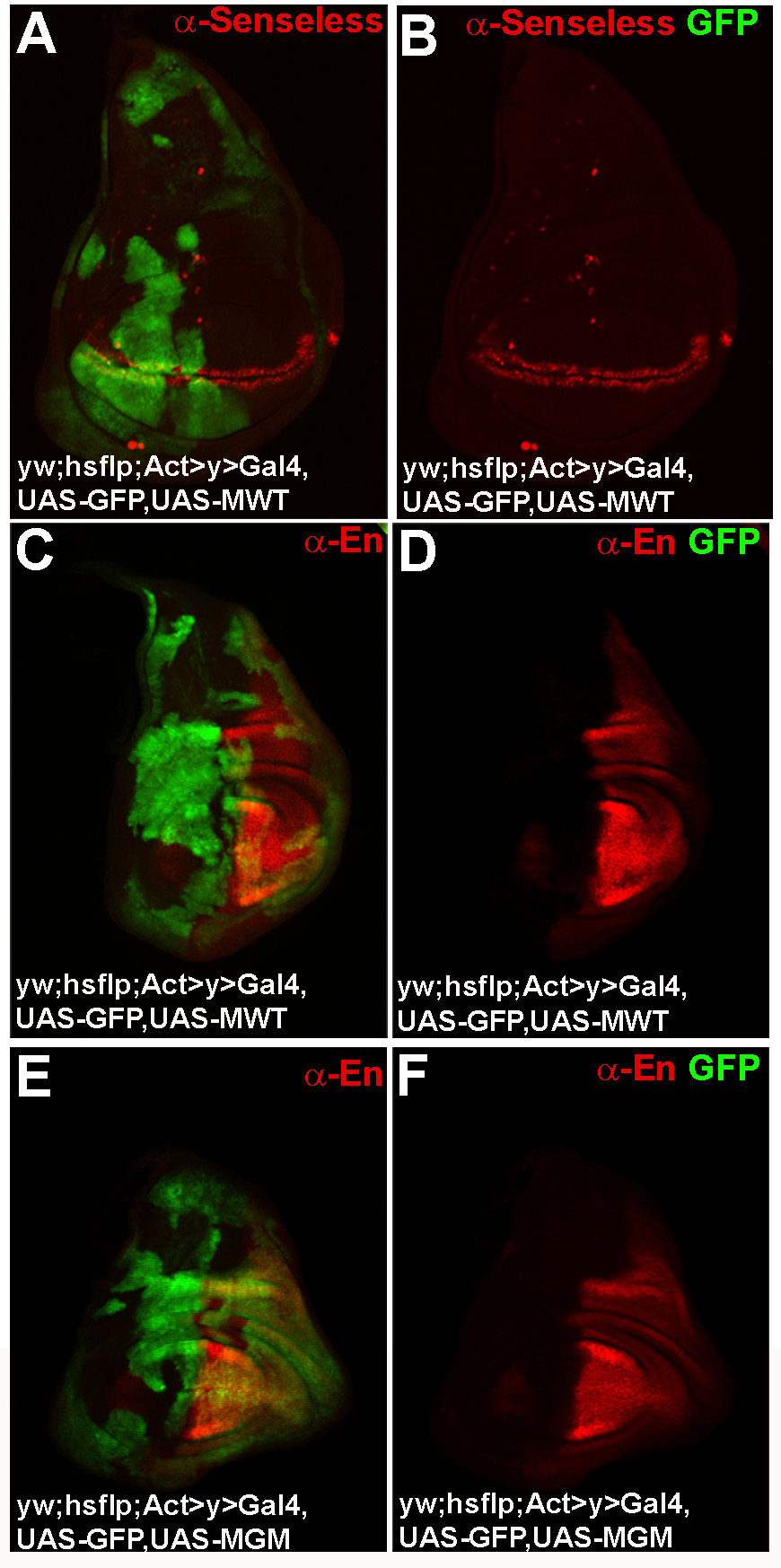

Supplement: Figure S5 — Clonal Analyses of Overexpressed Mad Proteins in Wing Discs. (A and B) Clonal expression of MWT (marked by GFP) does not increase Senseless expression along the presumptive wing margin. (C–D) MWT or MGM flp-out clones in the anterior compartment of wing discs do not cause ectopic expression of Engrailed protein (which is expressed only in the posterior compartment). These results also indicate that Hedgehog is not expressed in the anterior compartment within or around these clones, since ectopic En would reprogram cells in the anterior compartment to express Hedgehog [57]. (4.58 MB TIF) [file pone.0006543.s005.tif]

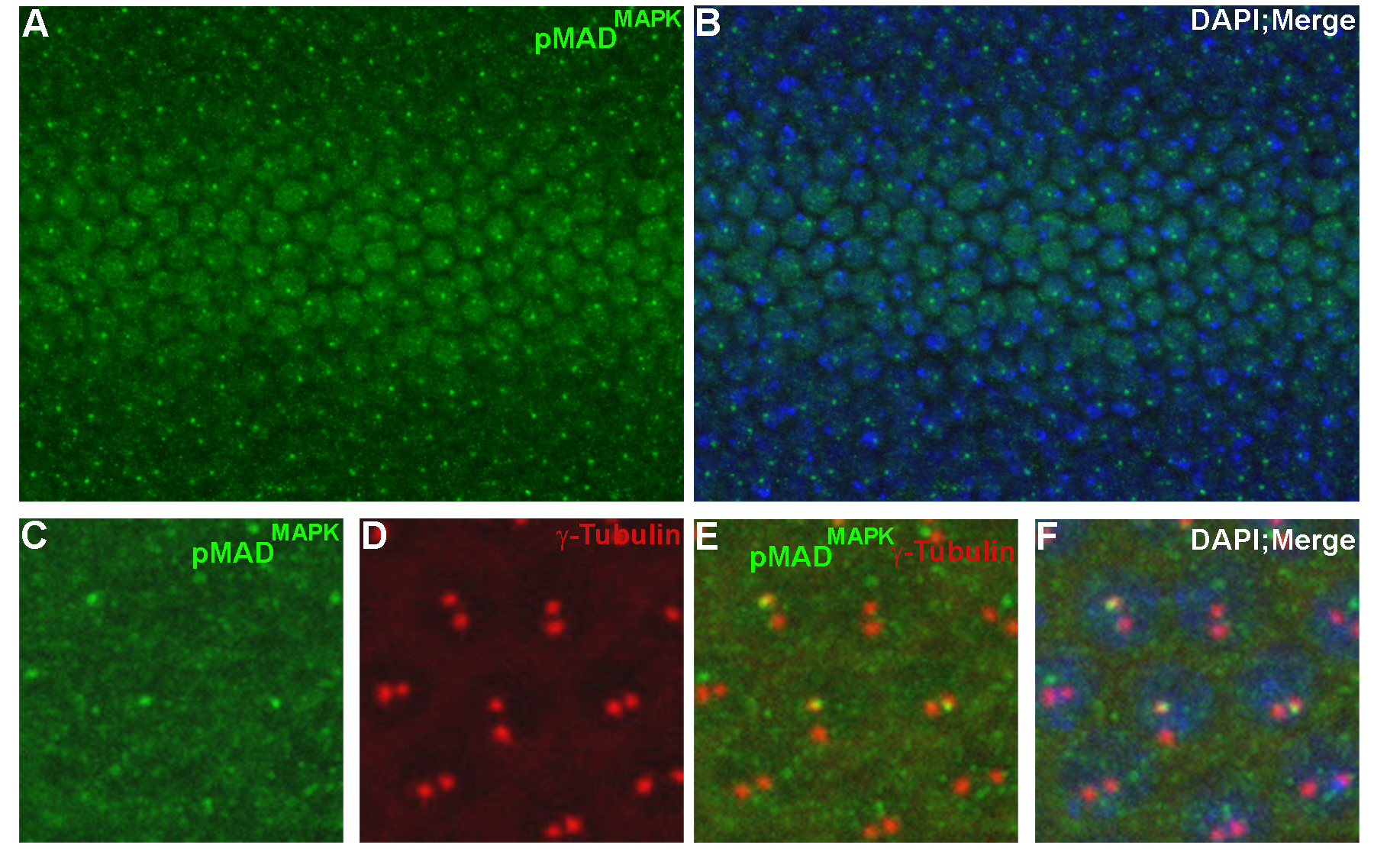

Supplement: Figure S6 — Asymmetric Immunostaining of pMadMAPK in Drosophila Cellular Blastoderm Cells. (A and B) Nuclear pMadMAPK visualized along a dorsal stripe. The nuclear pMadMAPK staining tracks pMadCter, which is dependent on Dpp signaling. A single bright cytoplasmic spot is apparent in most cells in stage 6 Drosophila embryos, which is seen in both nuclear and non-nuclear stained cells. (C–F) High power of a field of blastoderm cells, showing that the pMadMAPK spot is either adjacent or co-localizes with one of the centrosomes marked by γ-Tubulin. These cytoplasmic spots most likely represent Mad targeted for degradation to pericentrosomal proteasomes [6]. (6.17 MB TIF) [file pone.0006543.s006.tif]

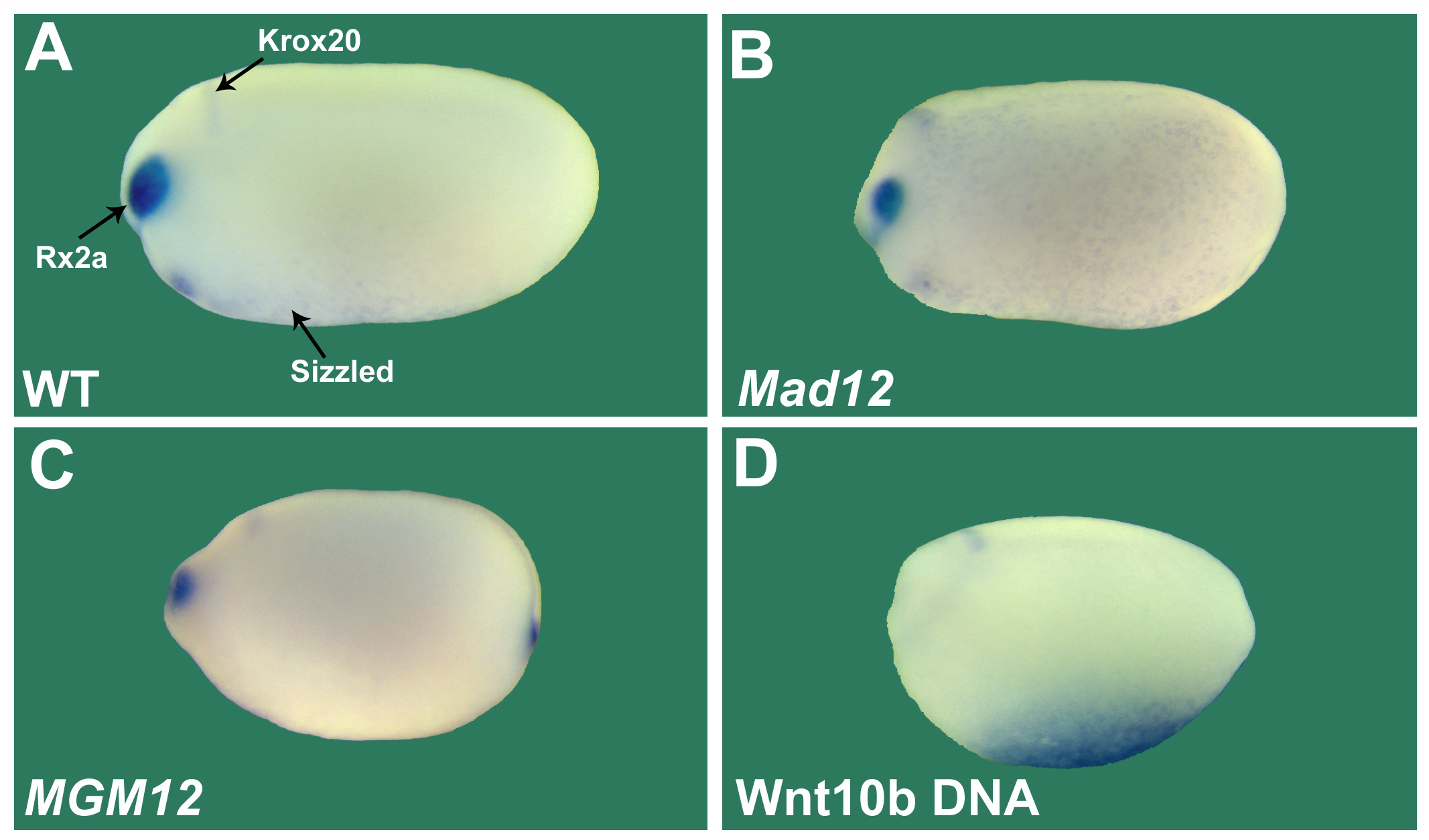

Supplement: Figure S7 — The Mad12 mutant, which lacks the C-terminal phosphorylation sites, retains posteriorizing activity in Xenopus, in particular when the GSK3 sites are also mutated. (A) Whole mount in situ hybridization of tail bud stage Xenopus embryos (n = 16). Embryos are stained for Rx2a (eye), Krox20 (Hindbrain) and Sizzled (Ventral/Belly). (B) Microinjection of Mad12 mRNA reduced the anterior head region of the embryo, indicated by decreased Rx2a expression (n = 15). (C) Elimination of the anterior head structures in MGM12 microinjected mRNAs (mutation of the GSK3 phosphorylation sites mimics Mad receiving a maximal Wg signal) resulted in a severely posteriorized embryo with almost complete loss-of Rx2a expression (n = 13). (D) Similar posteriorized phenotypes are generated when Wnt10b DNA is microinjected into Xenopus embryos. In addition to this, there is an increase in the expression of the BMP responsive gene sizzled, presumably because it affects the stability of endogenous Smad1/5/8 (n = 7). (1.34 MB TIF) [file pone.0006543.s007.tif]

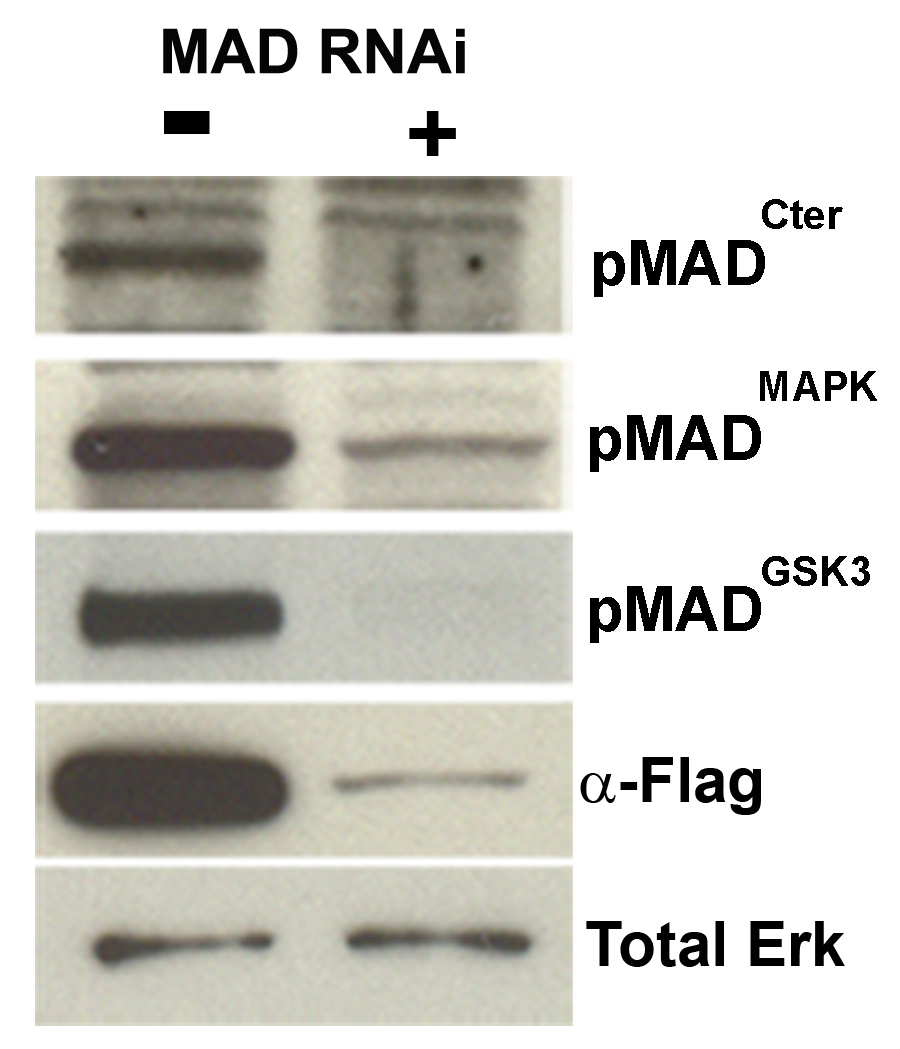

Supplement: Figure S8 — Expression of Mad RNAi in S2 Cells Specifically Inhibits Mad. Flag-tagged Mad was transiently co-transfected with empty vector (minus lane) or a construct expressing UAST Mad RNAi in Drosophila S2 cells co-transfected with a Gal4 plasmid (plus lane). Note that the different phosphorylated forms of Mad (pMadCter, pMadMAPK and pMADGSK3), as well as total Mad (α-Flag), were significantly reduced by co-expression of Mad RNAi. The expression of total Erk serves as control for equal loading. (2.86 MB TIF) [file pone.0006543.s008.tif]

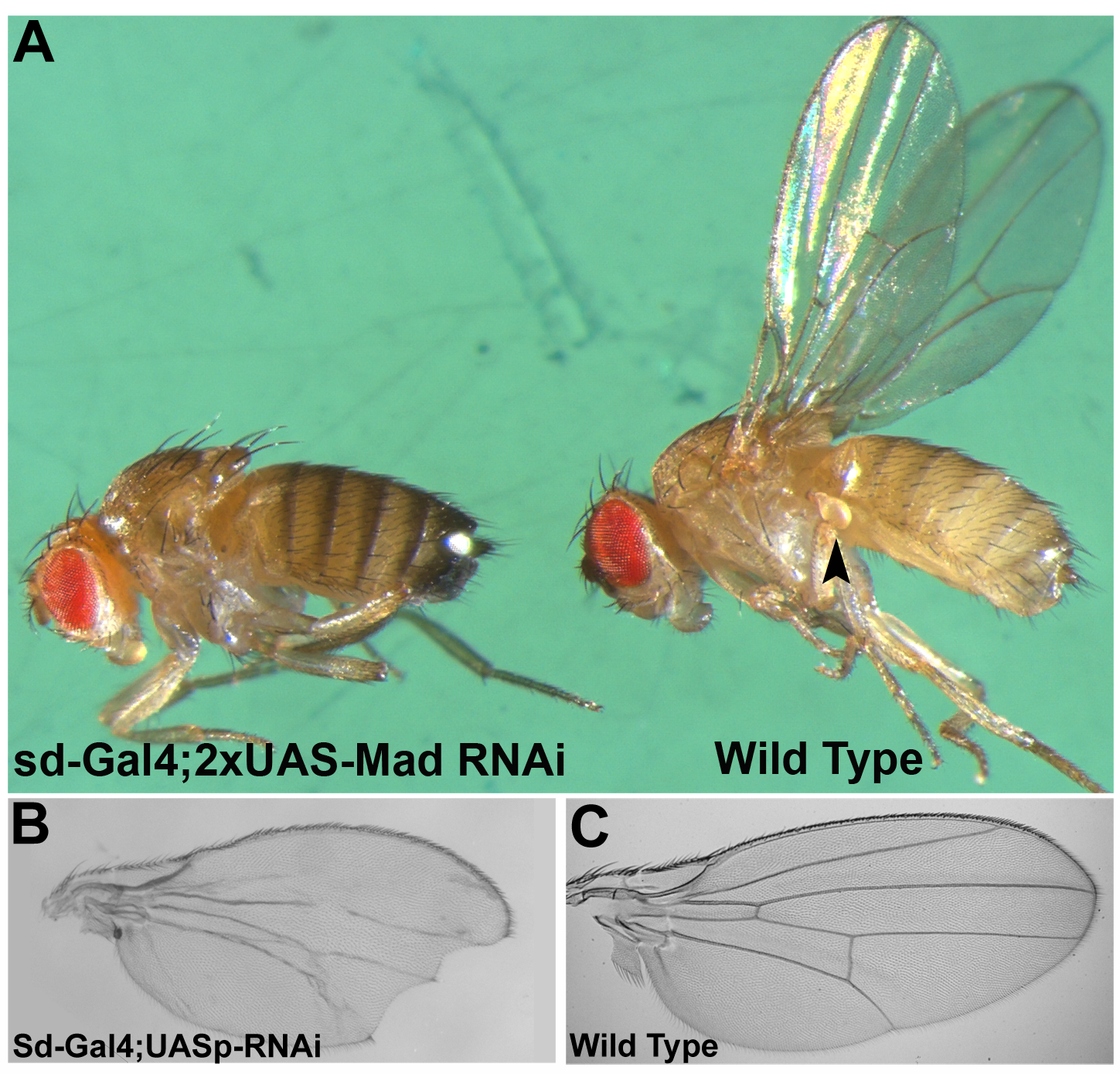

Supplement: Figure S9 — Mad RNAi Can Completely Block Wing and Haltere Development. (A) Loss of adult wings and halteres when two copies of UAS-Mad RNAi were driven with Scalloped-Gal4 at room temperature. Arrowhead indicates the location of the haltere in the wild-type fly. (B and C) Loss of wing margin accompanied by notching of the wing blade was found occasionally when UASp Mad RNAi (or UAST MAD RNAi, data not shown) was driven by scalloped-Gal4. These wing notches resemble a Wg loss-of function phenotype. Similar losses of margin tissue have previously been observed in weak genetic mutants in the Dpp/Mad pathway [32]. (6.07 MB TIF) [file pone.0006543.s009.tif]

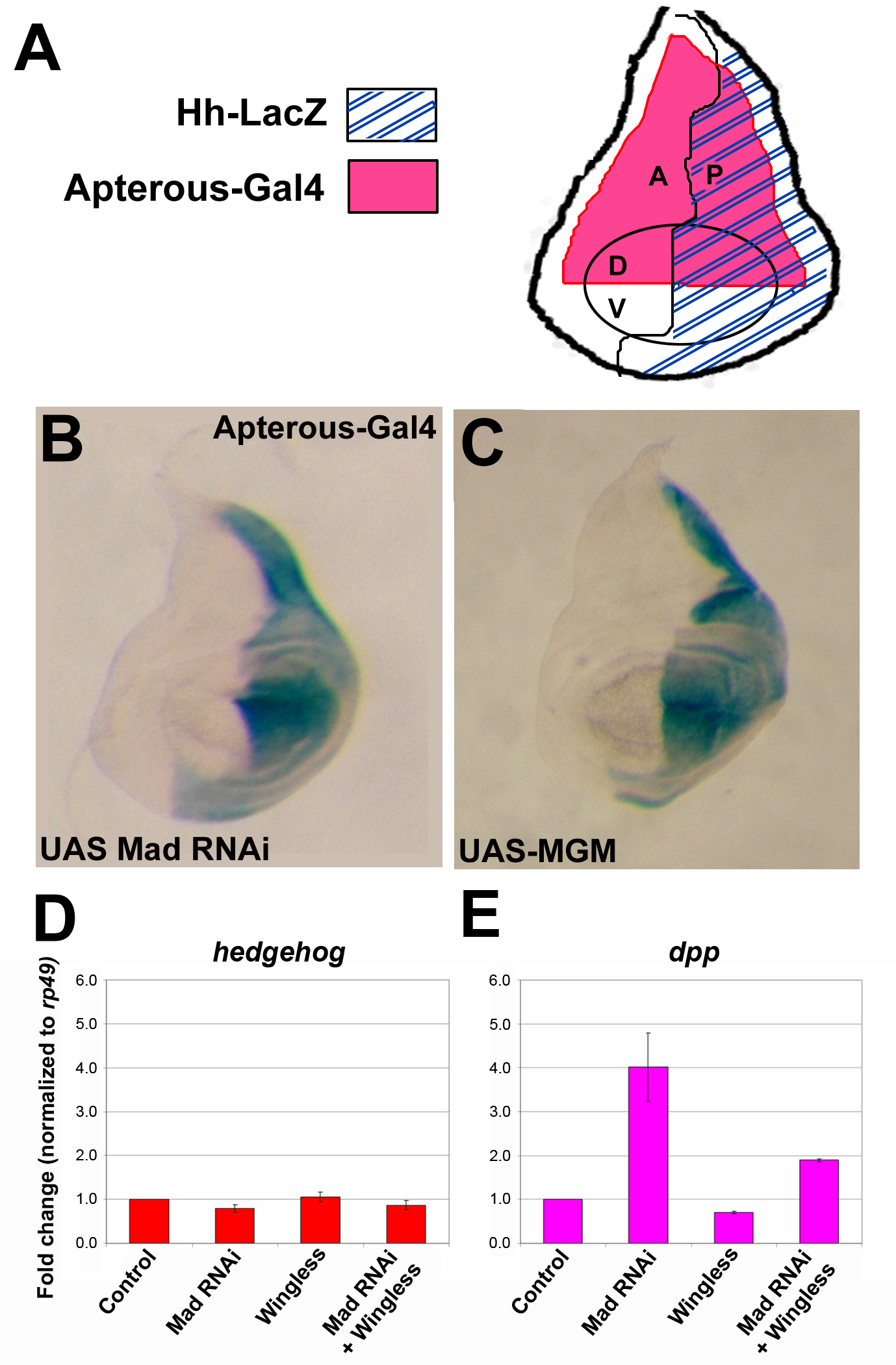

Supplement: Figure S10 — Mad RNAi does not Affect Hedgehog Expression. (A–C) Driving Mad RNAi or MGM with apterous-Gal4 in the dorsal compartment of the does not cause ectopic induction of a hedgehog-lacZ reporter in the anterior wing compartment. (D) Hedgehog mRNA levels are unaltered when Mad RNAi or Wg were overexpressed in the wing pouch using scalloped-Gal4. Expression also remained unchanged in wing discs when both Mad RNAi and Wg were co-expressed. (E) Dpp expression is increased when Mad is depleted in wing disc using scalloped-Gal4. However, this transcriptional increase in Dpp expression does not increase signaling because of the knockdown of Mad. Because Mad is depleted, these wings still display a Dpp loss-of function phenotype. Wg only fractionally decreased Dpp expression. These experiments serve as controls for the epistatic studies in Figure 4N–4Q, showing that the increase in marker genes caused by Wg, or the decrease caused by Mad RNAi, can not be explained by changes in Hedgehog or Dpp levels. (8.72 MB TIF) [file pone.0006543.s010.tif]

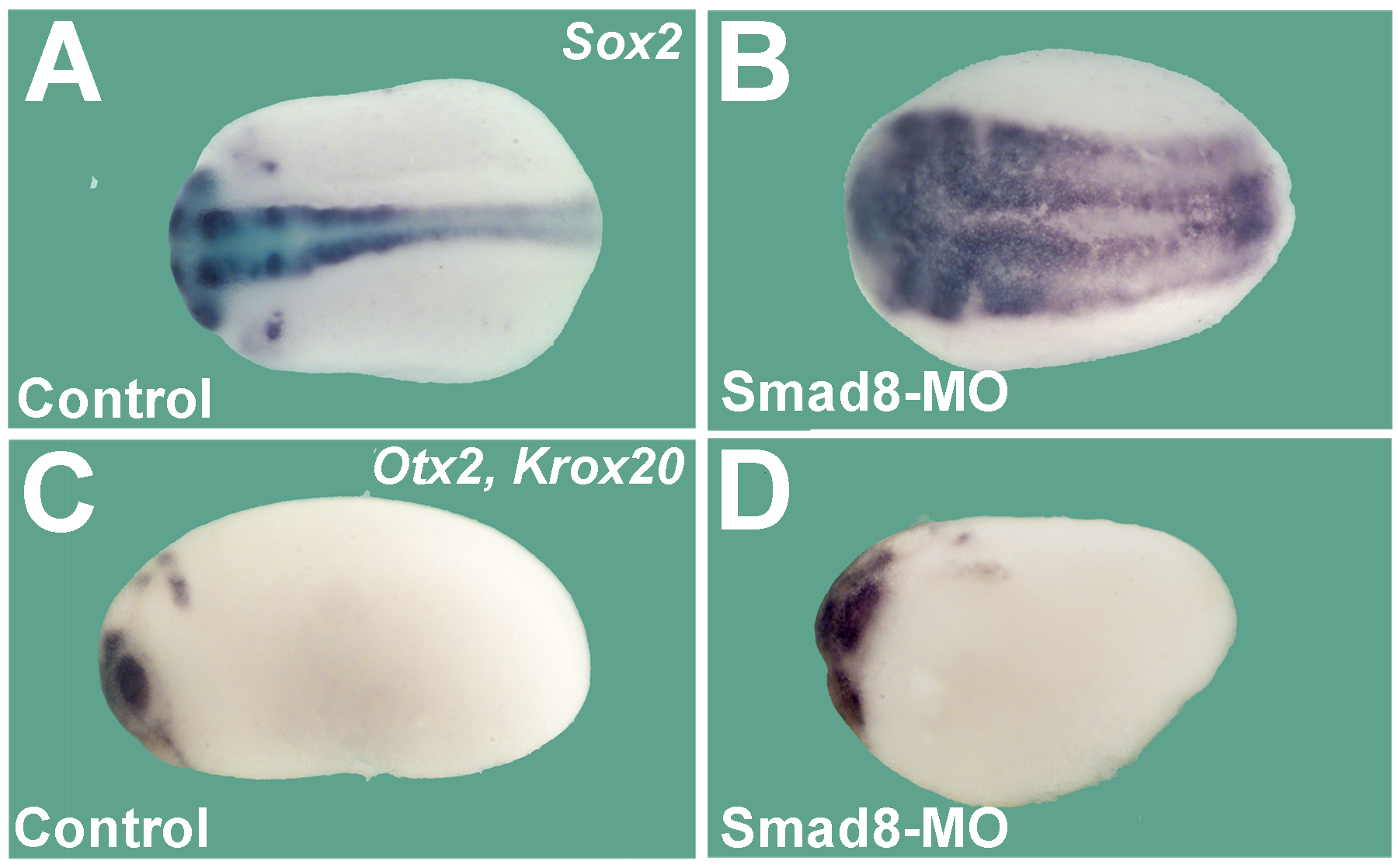

Supplement: Figure S11 — Knockdown of xSmad8 Dorsalizes Xenopus Embryos. (A) Whole-mount in situ hybridization for the pan-neural marker Sox2 in an uninjected control embryo, stage 22, dorsal view. (B) Injection of xSmad8 morpholino (0.5 mM, 4 nl injected four times radially) leads to expansion of the neural plate. (C) Control embryo stained for Otx2 (forebrain and midbrain marker) and Krox20 (hindbrain, rhombomeres 3 and 5), lateral view. (D) Smad8-depleted embryos are dorsalized (anti-BMP phenotype) and show expansion of head structures. It should be mentioned here that the original depletion of Xenopus laevis Smad8 by Miyanaga et al [30] yielded a very different result, namely apoptosis via activation of caspases. However, it should be noted that their methods for depletion were different. They used DNA oligonucleotides to deplete Smad8 transcripts in oocytes that were then subjected to maternal transfer and fertilization. We used morpholino oligos (of a different sequence) injected at the 4-cell stage, and therefore the depletion of maternal transcripts must have been less extensive. This explains why we did not observe apoptosis, but instead dorsalization (anti-BMP phenotype) of the embryo. The morpholino described here provides a useful reagent for knockdown of the maternal Xenopus laevis BMP-Smad. Smad8 probably corresponds to the homolog of zebrafish Smad5 [10] and is therefore referred to below as Smad5/8. (6.06 MB TIF) [file pone.0006543.s011.tif]

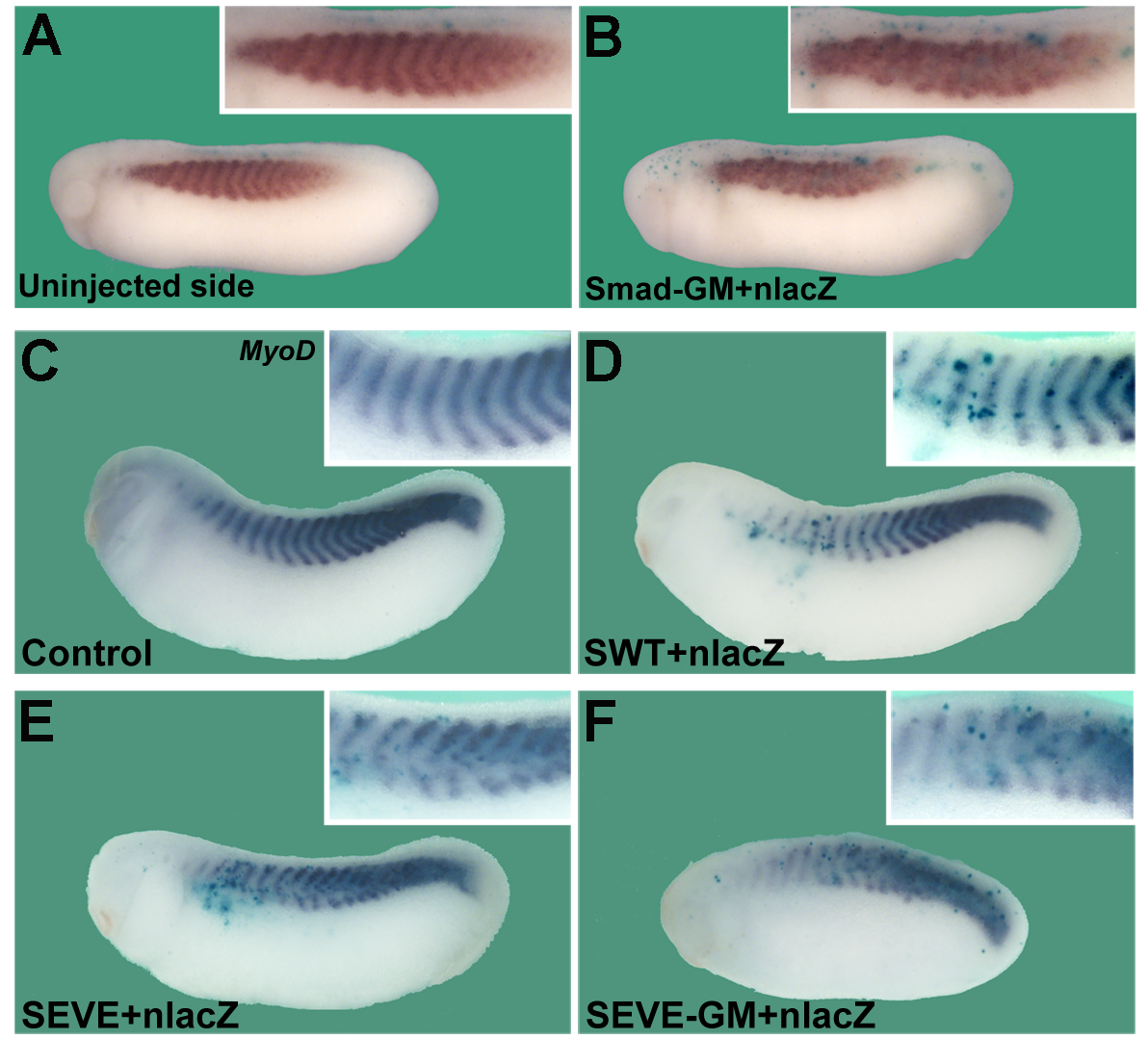

Supplement: Figure S12 — GSK3-resistant Activated Forms of Smad1 Disrupt Segmentation in Xenopus Embryos. (A and B) Immunostainings for myosin light chain showing loss of segmental borders in somites in the injected side (B) of a Xenopus embryo (stage 26), compared to the uninjected side. C2 or C3 blastomeres were injected with 100 pg of hSmad1 resistant to GSK3 phosphorylation [5] and 50 pg of nuclear LacZ mRNA (n = 32/42). (C–F) In situ hybridizations for the somite marker MyoD shows a disruption of the segmental pattern in Xenopus embryos (stage 30) injected with activated forms of Smad1. Uninjected control embryos express MyoD in the somitic segments in a typical chevron shape (n = 27). Overexpression of Smad1 wild-type mRNA (SWT) does not change this pattern (n = 21). An activated mutant of Smad1 that has phospho-mimetic amino acid substitutions on the C-terminus (the two most c-terminal serines mutated into glutamic acids, designated “SEVE” mutant [5]) displays mild disruptions of the somite pattern (n = 9/12), while the same phospho-mimetic form of Smad1 with an additional mutation in the GSK3-phosphorylation site in the linker (named “SEVE-GM”) exhibits strongly impaired segmentation (n = 14/18). Nuclear LacZ marks the injected cells. (3.89 MB TIF) [file pone.0006543.s012.tif]
